# Systemic Inflammatory Factors and Neuropsychiatric Disorders: A Bidirectional Mendelian Randomization Study

**DOI:** 10.1002/brb3.70478

**Published:** 2025-04-09

**Authors:** Hao Wang, Ziji Cheng, Zixuan Xu, Min Wang, Xingyang Sun, Wen Liu, Jingtian Wang, Qian Yang, Tuo Zhang, Jie Song, Yanjun Du, Xiaoming Zhang

**Affiliations:** ^1^ College of Acupuncture‐Moxibustion and Orthopedics Hubei University of Chinese Medicine Wuhan China; ^2^ School of Basic Medicine Hubei University of Chinese Medicine Wuhan China; ^3^ Department of Anatomy, College of Chinese Integrative Medicine Shanghai University of Traditional Chinese Medicine Shanghai China

**Keywords:** Mendelian randomization, neuropsychiatric disorders, systemic inflammatory factors

## Abstract

**Background:**

The present study employed Mendelian randomization to scrutinize the causal connections that may exist between 91 distinct inflammatory markers and six neuropsychiatric disorders, namely Alzheimer's disease (AD), multiple sclerosis (MS), Parkinson's disease (PD), anxiety disorders (ANX), depressive disorders (DEP), and unexplained encephalopathy (UE).

**Discussion:**

The methodology utilized involved the standard inverse variance weighting method within a two‐sample, two‐way Mendelian randomization framework and integrated statistics from genome‐wide association studies. To ascertain the robustness of the identified causal associations, sensitivity analyses were performed with the aid of the MR‐Egger method and the weighted median test.

**Conclusion:**

The results revealed that 14 distinct systemic inflammatory modulators are potentially causally linked to the risk of developing various neuropsychiatric disorders. Specifically, five were associated with AD, eight with ANX, six with DEP, and one with UE. However, the causal associations involving systemic inflammatory markers with PD and MS require further investigation, particularly with the identification of additional significant genetic variants. Furthermore, the concentration levels of 33 systemic inflammatory factors could be modulated by the occurrence of neuropsychiatric conditions, indicated by this study. These include five affected by AD, eight by PD, six by MS, 12 by ANX, five by DEP, and five by UE.

## Graphical abstract

This MR study investigated the bidirectional causal relationships between 91 inflammatory factors and 6 neuropsychiatric disorders. The results revealed that: The concentration levels of 14 inflammatory factors exhibited causal effects on 4 disorders; 6 disorders causally influenced the concentration levels of 33 inflammatory factors. Key findings: CD40L, IL‐12B, Casp‐8, and IL‐18R1 were causally associated with the risk of both ANX and DEP. PD and ANX influenced the concentration of S100‐A12; MS and ANX affected CD40L levels; ANX and DEP altered the concentration of IL‐17C.

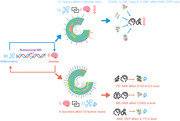

## 1 Introduction

Neuropsychiatric disorders encompass a range of conditions that originate from psychological symptoms of emotional distress or abnormal behavioral expressions. Given the nebulous distinction between neuropsychiatric and mental disorders, coupled with the high prevalence of comorbidities, the concept of neuropsychiatric disorders offers a holistic perspective on mental and neurological conditions. This category currently includes Alzheimer's disease (AD), multiple sclerosis (MS), Parkinson's disease (PD), anxiety (ANX), depression (DEP), unexplained encephalopathy (UE), and six other disorders. Previous research exploring the link between inflammatory factors and neuropsychiatric disorders has yet to definitively establish a causal relationship between these elements. Studies have primarily investigated the association between 41 systemic inflammatory factors and various disorders. In the current study, we selected the most recent summary statistics, incorporating 91 systemic inflammatory factors, and applied Mendelian randomization (MR) research methods. This approach aims to shed light on the cause‐and‐effect relationship between systemic inflammatory factors and neuropsychiatric disorders.

AD represents the predominant type of dementia, constituting 60%–80% of diagnosed instances. Inflammation has emerged as a significant contributor to the pathogenesis of AD (Heppner et al. 2015). Evidence suggests that alterations in systemic inflammatory levels may accelerate the disease's progression and are associated with modifications in the blood–brain barrier (Darweesh et al. 2018; Lai et al. 2017). Furthermore, a systemic immune challenge alone has been shown to elicit neurological symptoms akin to those of AD (Krstic et al. 2012). However, the question of whether systemic inflammation is the driving force or merely an outcome of AD remains unresolved, with observational studies yielding conflicting results in meta‐analyses (Holmes et al. 2009; Varatharaj and Galea 2017).

Recent research has also implicated inflammatory processes in the pathogenesis of PD (Hirsch and Hunot 2009; Houser and Tansey 2017; Kline et al. 2021; Wang et al. 2015). Within the inflammatory cascade, microglia are posited to be instrumental, potentially instigating the demise of dopaminergic neurons. After being activated, microglial cells with a toxic phenotype release pro‐inflammatory cytokines, which are detrimental to dopaminergic neurons, for instance, IL‐6 and TNF‐α. Additionally, mitochondrial stress can initiate inflammation through the release of damage‐associated molecular patterns (Sliter et al. 2018). Studies have observed elevated levels of IL‐1β, IL‐6, and IL‐10, which are kinds of pro‐inflammatory cytokines, and TNF‐α, in individuals with PD compared to controls, implicating that peripheral tissues may contribute to the origin of inflammation (Dowlati et al. 2010).

MS is defined as a disorder that is both inflammatory and neurodegenerative, primarily impacting the central nervous system (CNS) (Byrne, Yang, and Wray 2017). The typical course of MS involves intermittent inflammatory disease activity, often progressing to a state of cumulative neurologic deficit (Jacques 2015). The variability in the course of MS among patients underscores the gaps in our understanding of the factors that influence this progression. Innate immune dysfunction has been proposed as a potential mechanism underlying DEP disorders and other psychiatric disorders, offering new avenues for treatment (Dantzer et al. 2008; Nusslock and Miller 2016). Low‐grade systemic inflammation has been observed in 21%–34% of patients with depression, as indicated by elevated C‐reactive protein inflammatory factor concentrations and increased levels of IL‐6 and other inflammatory cytokines (Dowlati et al. 2010; Haapakoski et al. 2015; Orlovska‐Waast et al. 2019). Trials, which are both randomized and controlled, are in progress to evaluate the efficacy of anti‐inflammatory medications as potential treatments for depression.

ANX disorders share genetic and clinical overlap with DEP, and anxiety symptoms are now included in the diagnostic criteria for major depressive disorder (MDD). Initial evidence from case–control studies suggests a potential association between inflammation and generalized anxiety disorder (GAD), although the current study's results are inconclusive. Prospective studies provide more substantial evidence that inflammation may increase following the onset of an anxiety disorder. UE encompasses a range of encephalopathic disorders characterized by abnormal brain function or structure, with the specific cause remaining undetermined. This category includes non‐specific brain dysfunction, unclassified neurological disorders, structural brain abnormalities, and other neurological disorders.

MR represents an advanced and reliable method, leveraging genetic variants as instrumental variables, facilitating the exploration of causal relationships between exposures and outcomes of interest (Choi et al. 2019). By exclusively examining the genetically determined component of the exposure, MR mitigates the risk of inverse associations between the exposure and outcome that arise from the outcome variant but are not influenced by the genetic variant. Moreover, MR estimates are impervious to confounding factors, as genotypes, once established at conception, are inherently stable and not subject to change (Byrne et al. 2017).

Capitalizing on recent advancements in systemic inflammatory modulators research, including GWAS‐derived data for 91 circulating cytokines and six neuropsychiatric disorders (AD, PD, MS, ANX, DEP, and UE), we conducted bidirectional two‐sample MR analyses. Our approach comprised two distinct analytical phases: (1) forward‐direction MR: using inflammatory modulator‐associated single nucleotide polymorphisms (SNPs) (genetic instruments for cytokines) derived from blood‐based GWAS, we estimated their causal effects on neuropsychiatric disorders and (2) reverse‐direction MR: using neuropsychiatric disorder‐associated SNPs (genetic instruments for AD/PD/MS/ANX/DEP/UE) from independent GWAS, we assessed their causal impacts on systemic inflammatory modulators.

This bidirectional framework specifically aimed to (1) quantify the causal influence of genetically regulated inflammatory factors (cytokines/chemokines) on neuropsychiatric disorder susceptibility, (2) evaluate potential feedback effects whereby neuropsychiatric disorders alter inflammatory mediator levels, and (3) identify cytokines demonstrating bidirectional pleiotropy (e.g., mediators acting as both cause and consequence of neurological conditions). By rigorously separating genetic instruments (SNPs) from exposure/outcome variables (inflammatory markers vs. neurological disorders) across independent cohorts, this study clarifies immune‐neural axis interactions. Findings may reveal cytokine signatures for early neuropsychiatric diagnosis and modifiable targets for immunomodulatory therapies.

## 2 Methods

### 2.1 Study Design

**FIGURE 1:**
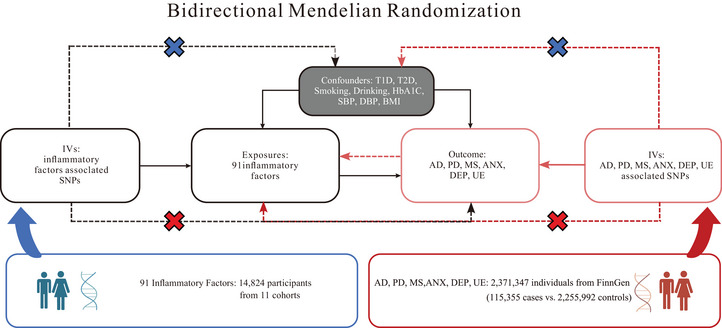
Graphical representation of the MR assumptions.

With the purpose of evaluating the causal link between systemic inflammatory cytokines and neuropsychiatric disorders, a two‐way MR study was performed. A detailed study layout is provided. We utilized data from previously published studies, and all necessary ethical approvals were obtained from the relevant institutional ethics committees. Valid instrumental variables, which we referred to as SNPs in this study, were selected by meeting the below three key criteria: (1) the SNPs must demonstrate a robust correlation with the exposure variable; (2) the SNPs should be free of confounding factors that might have an influence on both the exposure and outcome variables; and (3) the SNPs’ relationship with the outcome variable must be exclusively through their mediation of the exposure variable, with no direct impact on the outcome (Choi et al. 2019) (Figure 1).

### 2.2 Data Sources

We retrieved summary statistics for 91 genes involved in systemic inflammation from a genome‐wide association study (GWAS), which Zhao et al. (2023) most recently performed, involving 14,824 participants from 11 cohorts. The study used the Olink Targeted Inflammation Panel to measure genome‐wide genetic data and plasma proteomic data (Zhao et al. 2023).

The most recent summary statistics from the GWAS for neuropsychiatric disorders have been provided by the FinnGen research program, accessible at www.finngen.fi/fi. FinnGen, a pioneering initiative in genomics and personalized medicine, represents a substantial public–private collaboration. This project involves the collection and analysis of genomic and health‐related data from a cohort of 500,000 Finnish biobank participants. Its primary objective is to elucidate the genetic underpinnings of various diseases.

In our study, we have employed the latest dataset released by FinnGen, which encompasses the data of genome‐wide or genome‐wide meta‐analysis. This dataset includes information on the following conditions: AD with 15,617 cases and 396,564 controls, PD with 4681 cases and 407,500 controls, MS with 2409 cases and 408,561 controls, ANX with 44,663 cases and 301,879 controls, DEP with 47,696 cases and 359,290 controls, and UE with 289 cases and 382,198 controls.

### 2.3 Instrumental Variables

To identify instrumental variables, also known as SNPs, from the data of GWAS associated with the exposure variables, we meticulously established a stringent genome‐wide *p*‐value threshold, which was set at 1 × 10^−5^. This threshold was crucial in ensuring that only the most significant SNPs were selected for further analysis. Additionally, we also set a linkage disequilibrium (LD) threshold of *r* ^2^ < 0.01, which helped us to identify SNPs that were not in close proximity to each other, thus providing us with more reliable and independent genetic markers. Furthermore, to ensure that the selected SNPs were sufficiently distant from each other, we established a specific distance criterion of 5000 kb between each SNP. Due to the absence of allele frequencies in the GWASs of systemic inflammation modifiers, we were unable to confirm whether these SNPs exhibited consistency in the direction of their associations with both the exposure and outcome variables. Consequently, we excluded palindromic SNPs. To gauge the potency of the SNP–exposure relationship as an instrumental variable, we determined the average f‐statistic for each SNP. This involved approximating the f‐statistic as the square of the beta coefficient divided by the variance of association between SNP and exposure.

### 2.4 Statistical Analysis

The method of standard inverse variance weighting (IVW) with fixed effects was utilized for estimating causal effects in our two‐way, two‐sample MR analyses. The IVW method is a validated approach that utilizes inverse variance weighting of SNP (inflammatory factor)–outcome associations. In this method, the causal relationship between exposure and outcome can be consistently achieved, provided that all selected instrumental variables (IVs) are valid and there is no horizontal pleiotropy. However, if the IVs do not meet the “no horizontal pleiotropy” assumption, the estimated causal effect may be substantially biased. To address this concern, sensitivity analyses were undertaken with the application of two extra MR methods: (1) the weighted median (WM) method, which can provide reliable estimates when as many as 50% of the IVs are not perfectly valid, and (2) a suite of sensitivity analyses to examine the heterogeneity and pleiotropic effects of the causal relationships identified by the aforementioned methods. These sensitivity analyses included MR‐Egger, Weighted mode, MR pleiotropy RESidual sum Object, WM estimator, and simple mode. Furthermore, we assessed the heterogeneity among IVs using Q statistic, where *p*< 0.05 was taken as a sign of substantial heterogeneity (Verbanck et al. 2018). The false discovery rate method for threshold correction was employed to accommodate for multiple testing. Correlations with *p*‐values below 0.05 were deemed indicative of potential causality. R version 4.3.2, in conjunction with the specialized software package “TwosampleMR,” was used for all MR analyses.

## 3 Results

### 3.1 The Impact of Systemic Inflammatory Regulatory Factors on Neuropsychiatric Disorders

We aimed to determine the causal link between systemic inflammation and neuropsychiatric conditions by utilizing the IVW approach. Additionally, MR Egger, simple mode, Weighted Median, and WM were used for comprehensive analysis. Supporting Information 1 provides a detailed account of the outcomes of all these analyses.

By the IVW methodology, we observed that decreased Neurturin （NRTN ） levels were linked to an increased onset of AD ((OR): 0.902, 95% (CI): 0.835–0.974, *p*: 0.008). Conversely, elevated levels of S100‐A12, IL‐33, TRAIL, and TRANCE were found to correlate positively with AD development (OR: 1.09, 95% CI: 1.01–1.177, *p*: 0.026; OR: 1.091, 95% CI: 1.007–1.181, *p*: 0.033; OR: 1.057, 95% CI: 1.004–1.113, *p*: 0.034; OR: 1.052, 95% CI: 1.002–1.104, *p*: 0.040). What is more, no indication of horizontal pleiotropy was observed for NRTN, S100‐A12, IL‐33, TRAIL, and TRANCE according to the MR‐Egger intercept, with respective *p*‐values of 0.188, 0.629, 0.208, 0.326, and 0.188 (Figure 3). In addition, the *Q*‐values derived from both the MR‐Egger and IVW tests suggested no significant heterogeneity for NRTN, S100‐A12, IL‐33, TRAIL, and TRANCE (all *p*‐values were greater than 0.05).

Through the application of IVW technique, we uncovered that IL12B, VEGFA, Casp‐8, TNFRSF9, IL18R1, and OPG in reduced level were linked to an ascended risk of developing ANX (OR: 0.974, 95% CI: 0.938–1.012, *p*: 0.010; OR: 0.994, 95% CI: 0.961–1.029, *p*: 0.015; OR: 0.958, 95% CI: 0.914–1.005, *p*: 0.018; OR: 0.986, 95% CI: 0.953–1.021, *p*: 0.022; OR: 0.995, 95% CI: 0.958–1.033, *p*: 0.023; OR: 0.989, 95% CI: 0.959–1.02, *p*: 0.024, respectively). Conversely, higher levels of CD40L and Interleukin‐10 Receptor Alpha (IL10RA) were linked to a raised risk (OR: 1.037, 95% CI: 1.007–1.068, *p* < 0.001; OR: 1.044, 95% CI: 1.006–1.082, *p*: 0.037). The MR‐Egger intercept and *Q*‐values did not indicate the presence of horizontal pleiotropy for the factors (*p*: 0.142 _ CD40L, *p*: 0.194 _ IL12B, *p*: 0.119 _ VEGFA, *p*: 0.426 _ Casp‐8, *p*: 0.827 _ TNFRSF9, *p*: 0.823 _ IL18R1, *p*: 0.987 _ OPG, *p*: 0.923 _ IL10RA) (Figure 3). In addition, both of the *Q*‐values indicated that no significant heterogeneity was found in CD40L, IL12B, VEGFA, Casp‐8, TNFRSF9, IL18R1, OPG, and IL10RA (all *p*‐values were greater than 0.05).

Utilizing the IVW approach, we discovered a positive correlation between reduced levels of CD40L, IL12B, IL18R1, and the start of DEP, with OR0.994 (95% CI: 0.961–1.029, *p*: 0.017), 0.98 (95% CI: 0.935–1.027, *p*: 0.044), and 0.997 (95% CI: 0.967–1.027, *p*: 0.046), respectively. Additionally, higher levels of VEGFA, ADA, and Casp‐8 were linked to a higher likelihood of DEP developing (OR: 1.035, 95% CI: 1.008–1.063, *p*: 0.011; OR: 1.014, 95% CI: 0.983–1.047, *p*: 0.026; OR: 1.005, 95% CI: 0.969–1.042, *p*: 0.048, respectively). The MR‐Egger intercept revealed no signs of horizontal pleiotropy associated with these factors (*p*: 0.468 _ VEGFA, *p*: 0.056 _ CD40L, *p*: 0.375 _ ADA, *p*: 0.52 _ IL12B, *p*: 0.375 _ IL18R1, *p*: 0.321 _ Caspase). Furthermore, both of the *Q*‐values did not indicate any evidence of substantial heterogeneity for VEGFA, CD40L, Adenosine Deaminase (ADA), IL12B, IL18R1, and Caspase (all *p*‐values were greater than 0.05).

**FIGURE 2:**
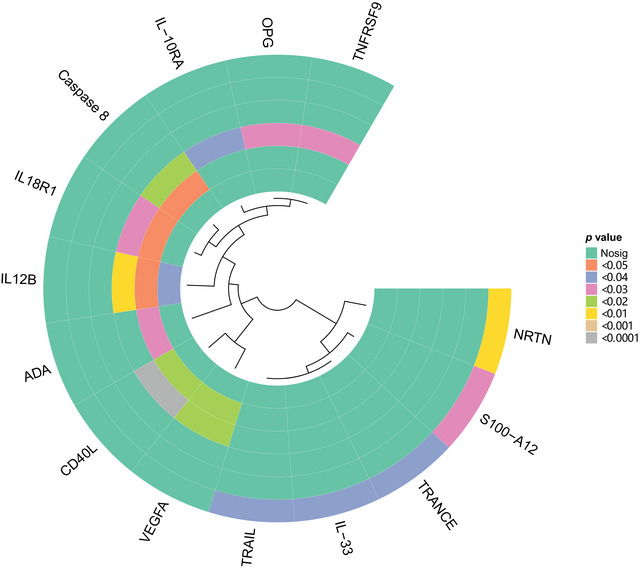
Hotspot map for forward MR analysis: Hotspot map depicting the effects of inflammatory regulatory factors on various neuropsychiatric disorders.

**FIGURE 3:**
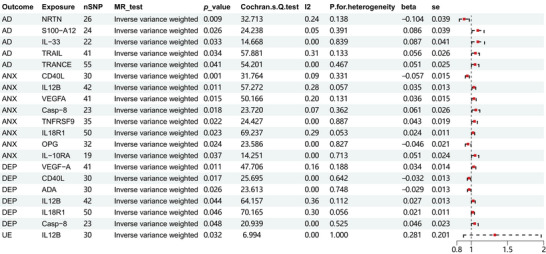
Forest plot for IVW analysis of forward MR analysis: Association of systemic inflammatory regulators with neuropsychiatric disorders through MR (encompassing genome‐wide significant SNPs). The OR and 95% CI in the figure represent the altered risk of neuropsychiatric disorders per 1‐standard deviation (SD) elevation in the systemic inflammatory regulator level.

By the IVW method, we detected a positive association between decreased IL12B levels and the incidence of UE (OR: 0.961, 95% CI: 0.703–1.313, *p*: 0.031). The MR‐Egger intercept did not provide evidence for the existence of horizontal pleiotropy in relation to IL12B (*p*: 0.969) (Figure 3). Additionally, the *Q*‐values displayed that IL12B was not significantly different (all *p*‐values were greater than 0.05). For a visual representation of these results, Figure 2 presents a hotspot map, and Figure 3 provides a forest plot of the findings.

**FIGURE 4:**
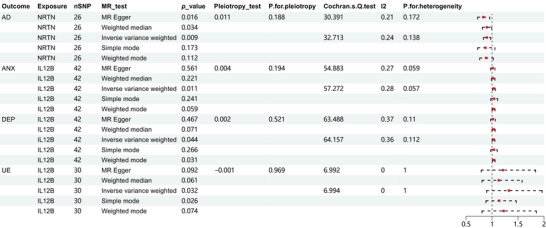
Forest plot of partial forward MR analysis: Forest plot of selected inflammatory factor proteins inflammatory factors associated with the potential occurrence of AD, ANX, DEP, and UE.

Figure 4 illustrates the NRTN proteins inflammatory factors that were strongly connected with the possibility of developing AD and the IL12B proteins inflammatory factors that were linked to the risk of developing ANX, DEP, and UE. The forest plot of all analyses is provided in Supporting Information 2 for further reference.

### 3.2 The Effect of Neuropsychiatric Disorders on the Levels of Regulatory Factors of Systemic Inflammation

We employed the same methodology to analyze whether neuropsychiatric disorders have an inverse effect on the levels of systemic inflammatory factors, with all relevant analyses detailed in Supporting Information 3.

Our study, conducted using the IVW method, demonstrated that the onset of AD was in a highly positive correlation with lower levels of CDCP1 and IL‐20 (OR: 0.917, 95% CI: 0.867–0.969, *p*: 0.002; OR: 0.959, 95% CI: 0.923–0.997, *p*: 0.033, respectively). Conversely, the elevated levels of Axin‐1, IL8, and MCP2 were linked to a heightened risk of AD (OR: 1.153, 95% CI: 1.037–1.282, *p*: 0.008; OR: 1.069, 95% CI: 1.014–1.127, *p*: 0.013; OR: 1.043, 95% CI: 1.005–1.083, *p*: 0.026, respectively). The MR‐Egger intercept and *Q*‐values suggested that there was no indication of horizontal pleiotropy for these factors (*p*: 0.751 for CDCP1, *p*: 0.939 _ Axin‐1, *p*: 0.442 _ IL8, *p*: 0.560 _MCP2, *p*: 0.191 _ IL‐20) (Figure 6). Furthermore, the *Q*‐values manifested that no significant heterogeneity was discovered for CDCP1, Axin‐1, IL8, MCP2, and IL‐20 (all *p*‐values were greater than 0.05).

Using the IVW approach, we found that the onset of PD was associated with the lower levels of SULT1A1, IL‐15RA, and PD‐L1 (OR: 0.899, 95% CI: 0.829–0.976, *p*: 0.011; OR: 0.912, 95% CI: 0.847–0.981, *p*: 0.014; OR: 0.888, 95% CI: 0.791–0.998, *p*: 0.047, respectively). Conversely, the higher levels of CXCL1, MIP1α, S100‐A12, EIF4EBP1, and CCL4 were connected to a higher risk of PD (OR: 1.129, 95% CI: 1.024–1.244, *p*: 0.015; OR: 1.088, 95% CI: 1.008–1.175, *p*: 0.031; OR: 1.101, 95% CI: 1.009–1.201, *p*: 0.032; OR: 1.107, 95% CI: 1.003–1.221, *p*: 0.043; OR: 1.063, 95% CI: 1.001–1.129, *p*: 0.048, respectively). The MR‐Egger intercept did not yield any indication of horizontal pleiotropy for these factors (*p*: 0.929 _ SULT1A1, *p*: 0.238 _ IL‐15RA, *p*: 0.786 _CXCL1, *p*: 0.143 _ MIP1α, *p*: 0.364 _ S100‐A12, *p*: 0.745 _ EIF4EBP1, *p*: 0.588 _ PD‐L1, *p*: 0.948 for CCL4) (Figure 6). Additionally, the *Q*‐values revealed no substantial heterogeneity for SULT1A1, IL‐15RA, CXCL1, MIP1α, S100‐A12, EIF4EBP1, PD‐L1, and CCL4 (all *p*‐values were greater than 0.05).

With the IVW approach, we discovered a decreased level of a positive correlation between the onset of MS and CD40L and Artemin (ARTN) (OR: 0.882, 95% CI: 0.791–0.983, *p*: 0.023; OR: 0.854, 95% CI: 0.739–0.988, *p*: 0.034, respectively). Conversely, MS onset showed a high level of a positive correlation with higher levels of MIP1α, TNF‐β, IL1α, and CXCL11 (OR: 1.199, 95% CI: 1.073–1.34, *p*: 0.001; OR: 1.478, 95% CI: 1.129–1.935, *p*: 0.004; OR: 1.194, 95% CI: 1.015–1.405, *p*: 0.033; OR: 1.155, 95% CI: 1.006–1.325, *p*: 0.040, respectively). No sign of horizontal pleiotropy for these factors was indicated by the MR‐Egger intercept (*p*: 0.140 _ MIP1α, *p*: 0.199 _ TNF‐β, *p*: 0.409 _ CD40L, *p*: 0.811 _ IL1a, *p*: 0.347 _ ARTN, *p*: 0.092 _ CXCL11) (Figure 6). Additionally, the *Q*‐values revealed no substantial heterogeneity for MIP1α, TNF‐β, CD40L, IL1α, ARTN, and CXCL11 (all *p*‐values were greater than 0.05).

The IVW approach revealed that the onset of ANX was positively correlated with lower levels of CD40L, OPG, IL17C, CCL19, and IL24 (OR: 0.95, 95% CI: 0.924–0.978, *p* < 0.001; OR: 0.952, 95% CI: 0.919–0.986, *p*: 0.007; OR: 0.956, 95% CI: 0.924–0.99, *p*: 0.011; OR: 0.959, 95% CI: 0.927–0.993, *p*: 0.017; OR: 0.95, 95% CI: 0.909–0.993, *p*: 0.024, respectively). Additionally, ANX onset was associated with the higher levels of M‐CSF1, CXCL11, TNFRSF9, IL10RA, IL12B, S100‐A12, and CD6 (OR: 1.057, 95% CI: 1.016–1.098, *p*: 0.006; OR: 1.042, 95% CI: 1.012–1.074, *p*: 0.006; OR: 1.048, 95% CI: 1.011–1.086, *p*: 0.011; OR: 1.035, 95% CI: 1.006–1.064, *p*: 0.017; OR: 1.059, 95% CI: 1.009–1.113, *p*: 0.021; OR: 1.041, 95% CI: 1.004–1.08, *p*: 0.03; OR: 1.032, 95% CI: 1.006–1.064, *p*: 0.047, respectively). The MR‐Egger bias parameter indicated no evidence of horizontal pleiotropy for these factors (*p*: 0.058 for CD40L, *p*: 0.516 _ M‐CSF1, *p*: 0.539 _ CXCL11, *p*: 0.179 _ OPG, *p*: 0.065 _ IL17C, *p*: 0.818 _ TNFRSF9, *p*: 0.348 _ CCL19, *p*: 0.996 _ IL10RA, *p*: 0.807 _ IL12B, *p*: 0.180 _ IL24, *p*: 0.900 _ S100‐A12, *p*: 0.738 for CD6) (Figure 6). Furthermore, both of the *Q*‐values revealed no substantial heterogeneity in CD40L, M‐CSF1, CXCL11, OPG, IL17C, TNFRSF9, CCL19, IL10RA, IL12B, IL24, S100‐A12, and CD6 (all *p*‐values were greater than 0.05).

Using the IVW approach, we observed a positive correlation between the onset of DEP and lower levels of IL17C, ADA, and MMP‐1 (OR: 0.955, 95% CI: 0.924–0.987, *p*: 0.006; OR: 0.967, 95% CI: 0.938–0.997, *p*: 0.033; OR: 0.957, 95% CI: 0.919–0.997, *p*: 0.036, respectively). Additionally, DEP onset was correlated with the elevated levels of VEGFA and CXCL10 (OR: 1.035, 95% CI: 1.01–1.061, *p*: 0.005; OR: 1.031, 95% CI: 1.008–1.055, *p*: 0.008, respectively). The MR‐Egger intercept suggested no evidence of horizontal pleiotropy for these factors (*p*: 0.745 _ VEGFA, *p*: 0.392 _ IL17C, *p*: 0.605 _ CXCL10, *p*: 0.834 _ ADA, *p*: 0.176 _ MMP‐1) (Figure 6). Furthermore, the *Q*‐values did not indicate significant heterogeneity for VEGFA, IL17C, CXCL10, ADA, and MMP‐1 (all *p*‐values were greater than 0.05).

**FIGURE 5:**
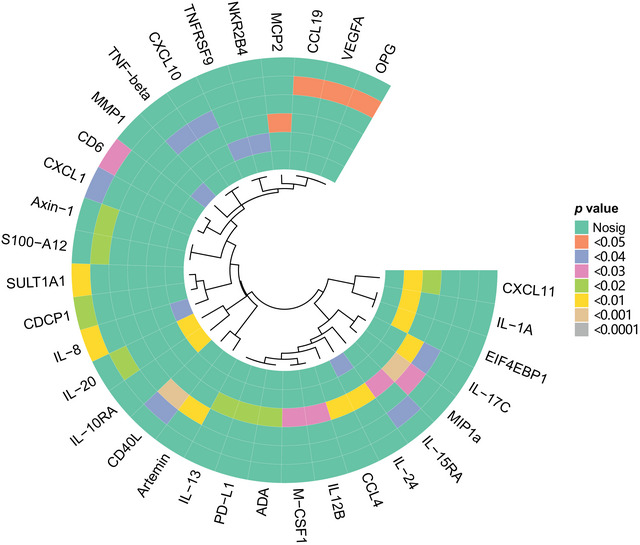
Hotspot map for reverse MR analysis: Hotspot map illustrating the effects of neuropsychiatric disorders on inflammatory regulatory factors.

**FIGURE 6:**
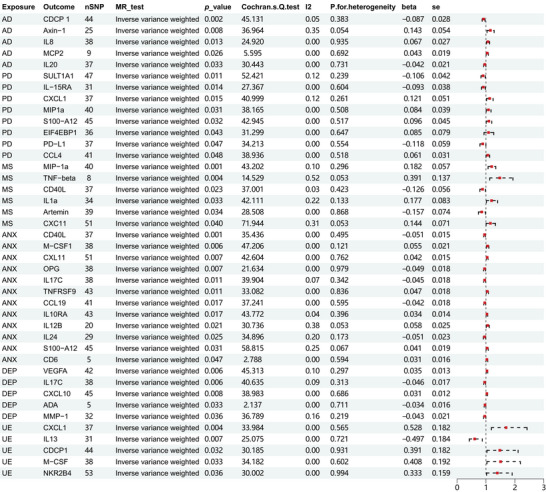
Forest plot of IVW analysis of reverse MR analysis: Correlation between systemic inflammatory regulators with neuropsychiatric disorders via MR (with genome‐wide significant SNPs). The OR and 95% CI in the figure depict the alteration in the concentration of systemic inflammatory regulators for each 1‐SD rise in the neuropsychiatric disorders.

Using the IVW approach, we found that the onset of UE was positively correlated with the reduced levels of IL13 (OR: 0.608, 95% CI: 0.424–0.873, *p*: 0.007). Conversely, the higher levels of CXCL1, CDCP1, M‐CSF1, and NKR2B4 were positively associated with the onset of UE (OR: 1.696, 95% CI: 1.188–2.421, *p*: 0.004; OR: 1.478, 95% CI: 1.034–2.113, *p*: 0.032; OR: 1.504, 95% CI: 1.033–2.191, *p*: 0.033; OR: 1.395, 95% CI: 1.022–1.905, *p*: 0.036, respectively). The MR‐Egger intercept did not indicate any evidence of horizontal pleiotropy for these factors (*p*: 0.110 _ CXCL1, *p*: 0.243 _ IL13, *p*: 0.200 _ CDCP1, *p*: 0.814 _ M‐CSF1, *p*: 0.512 _ NKR2B4) (Figure 6). Additionally, the *Q*‐values from both the MR‐Egger and IVW tests did not indicate significant heterogeneity for CXCL1, IL13, CDCP1, M‐CSF1, and NKR2B4 (all *p*‐values were greater than 0.05). For a visual representation of these results, Figure 5 presents a hotspot map, and Figure 6 provides a forest plot of the findings.

**FIGURE 7:**
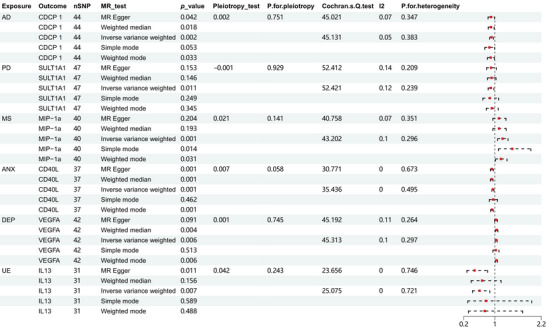
Forest plot of partial reverse MR analysis: A partial forest plot of systemic inflammatory factors affected by neuropsychiatric disorders.

Figure 7 displays a forest plot of systemic inflammatory factor proteins that were more significantly affected by neuropsychiatric disorders. For a comprehensive view of all forest plots, Supporting Information 4 is provided.

## 4 Discussion

We delved into the causal relationship of 91 systemic inflammatory regulators with six neuropsychiatric disorders through a bidirectional MR analysis. The results suggest that 14 unique systemic inflammatory regulators, with genetically predicted concentrations, could have a causal role in the onset of neuropsychiatric disorders. Notably, this is the first time a causal relationship has been established between systemic inflammatory regulatory factors and the development of AD and UE. Key findings include (1) NRTN at low levels, and S100‐A12, IL‐33, TRAIL, and TRANCE at high levels may influence the pathogenesis of AD; (2) IL12B, VEGFA, Casp‐8, TNFRSF9, IL18R1, and OPG at low levels and CD40L and IL10RA at high levels are related to the pathogenesis of ANX; (3) CD40L and IL10RA at high levels and CD40L, IL10RA at low levels are linked to the onset of DEP; (4) CD40L, IL12B, and IL18R1 at lower levels and VEGFA, ADA, and Casp‐8 at higher levels impact the onset of DEP; (5) IL12B at lower levels impact the onset of UE.

Furthermore, it is suggested that these neuropsychiatric disorders could have an impact on the concentration levels of 33 systemic inflammatory modulators. These include CDCP1 being less affected by AD, IL‐20by PD, Axin‐1, IL8, and MCP2 by PD, and SULT1A1, IL‐15RA, and PD‐L1 by PD. CXCL1, MIP1α, S100‐A12, EIF4EBP1, and CCL4 are more affected by PD. CD40L and ARTN are less affected by MS. MIP1α, TNF‐β, IL1α, and CXCL11 are affected by MS. CD40L, OPG, IL17C, CCL19, and IL24 are less affected by ANX, while M‐CSF1, CXCL11, TNFRSF9, IL10RA, IL12B, S100‐A12, and CD6 are more affected. DEP is less affected by IL17C, ADA, and MMP‐1 and more affected by VEGFA and CXCL10. IL13 is less affected by UE, while CXCL1, CDCP1, M‐CSF1, and NKR2B4 are more affected by UE.

Overall, both ANX and DEP have a causal relationship with the concentration levels of CD40L, IL12B, Casp‐8, and IL18R1. PD and ANX can both influence the concentration level of S100‐A12. MS and ANX will both impact the concentration level of CD40L. ANX and DEP can both affect the concentration level of IL17C.

This finding is of great significance: (1) it may provide us with new biomarkers for predicting the occurrence of mental disorders. If the changes in the concentrations of CD40L, IL12B, Casp‐8, and IL18R1 can be monitored in clinical, it may be possible to prevent psychiatric disorders. Early intervention is especially crucial, and these findings offer the possibility of developing new therapeutic targets. (2) It enables a better understanding of the connection in pathophysiological mechanisms between PD and ANX. S100‐A12 is a calcium‐binding protein that plays a role in processes such as inflammatory responses. The alteration in the concentration of S100‐A12 in patients with PD and ANX may imply the role of abnormal inflammatory responses or the function of glial cells in these diseases. (3) It reveals the potential common pathways or interaction mechanisms among these diseases. CD40L is an immune‐related molecule that plays a key role in the activation of the immune system and intercellular signal transduction. IL17C is a member of the interleukin family and is mainly involved in inflammatory responses and immune regulation. The changes in the concentrations of these molecules may reflect the important role of the immune system in neuropsychiatric disorders.

Systemic inflammatory modulators are known to exert neurodevelopmental effects from early in life (Jiang et al. 2018). They have been implicated in a range of adverse outcomes, including developmental abnormalities and functional deficits in the CNS. Examples include alterations in neonatal brain connectivity, cognitive impairment, decreased neurodevelopmental scores, cerebral inhibition defects, blood–brain barrier disruption, reduced functional connectivity of cortical lobes (Felger et al. 2016), and an increased risk of several neuropsychiatric disorders (Felger et al. 2016; Fernandes et al. 2016; Haddad et al. 2019; Miller and Raison 2016; Turkheimer et al. 2021). Despite the fact that the exact ways in which these modulators affect the body are not yet completely clear, there is ample evidence to suggest their involvement in the pathogenesis of neuropsychiatric disorders.

We discovered that NRTN and its concentration levels correlate with AD. NRTN, along with ARTN and PSPN, supports various neuronal populations in the CNS and also supports the survival and regulates the differentiation of several peripheral neurons. NRTN binds to the GFRα2 protein, forming a complex that leads to the transphosphorylation of two RET molecules at specific tyrosine residues in their structural domains, thereby initiating intracellular signaling. Clinical studies have indicated that NRTN, due to its role in regulating prominence formation, may contribute to the treatment of AD.

IL‐33, a pivotal player in the innate immune response, also governs the infiltration and activation of immune cells. It interacts with heterodimeric receptor complexes, triggering a signaling pathway that includes NF‐κB and myeloid differentiation factor 88 (MyD 88), ultimately leading to the selective activation of mast cells, type 2 T helper cells, neutrophils, and alternative‐activated macrophages. Additionally, genetic research has revealed that within the IL‐33 gene, three single nucleotide polymorphisms are linked to a decreased likelihood of developing AD (Chapuis et al. 2009; Fu et al. 2016). Studies have demonstrated that the precise modulation of the immune response is crucial for maintaining proper neurological function. The cytokine TRAIL, a part of the TNF superfamily characterized by its multifaceted functions, is involved in a range of processes both in the central nervous system and the peripheral system. Its roles include the initiation of cell death signaling, the modulation of immune responses, and the regulation of inflammatory reactions (Y. Huang et al. 2015). Two TRAIL‐activated death pathways have been identified: (1) the exogenous pathway, associated with the activation of Casp‐8, involves the assembly of the adaptor molecule Fas‐associated death domain protein (FADD), and (2) the mitochondrial apoptosis pathway, triggered by a signaling cascade mediated by the BH3‐interacting domain (Bid), culminates in the formation of a multimeric complex known as the “apoptosome.” This process involves mitochondrial outer membrane permeabilization, which activates effector caspases and releases cytochrome c, thereby facilitating the formation of the apoptosome (Sessler et al. 2013). In both peripheral lymphoid organs and the brain, TRAIL exerts a significant influence on neuronal survival in AD patients (Burgaletto et al. 2020). VEGFA is a critical mediator of angiogenesis and blood–brain barrier permeability, with a positive impact on blood–brain barrier dysfunction and depressive behaviors’ development. The findings of this study indicate that VEGFA is a critical factor in DEP development, as it contributes to promoting greater permeability within the blood–brain barrier (Dudek et al. 2020; Matsuno et al. 2022; Menard et al. 2017; Najjar et al. 2013). Elevated plasma levels of CD40L in the plasma of individuals suffering from DEP indicate that CD40L is influenced by DEP (Neubauer et al. 2013). The connection between CD40L levels and DEP is further strengthened by the notable discrepancies in CD40L levels observed between those who respond to treatment and those who do not in studies of depressed patients (Siddarth et al. 2023). ADA is a vital enzyme involved in the maturation and functioning of T lymphocytes. Studies indicate that the plasma activity of ADA is elevated in inflammatory diseases, which can elicit a cell‐mediated immune response. The activity level of ADA significantly influences the pathogenesis of DEP. The levels of IL18R1 were notably altered in response to DEP.

Concurrently, our research indicates that neuropsychiatric disorders may have an inverse impact on the levels of specific inflammatory regulators. Notably, ANX exhibits a strong association with CD40L, OPG, and IL12B and a significant correlation with ADA in the presence of DEP. Several of these cytokines have previously been identified as being associated with neuropsychiatric disorders. However, observational studies can be influenced by confounding variables, making it challenging to establish causal relationships.

By concentrating on alterations in the concentration levels of genetically determined inflammatory regulators, we discovered that changes in the levels of these inflammatory factors might represent a self‐protective mechanism of the body. This mechanism aims to mitigate inflammation and prevent the progression of neurological pathology through adjustments in the concentration of inflammatory factors. These inflammatory regulatory factors are potential candidates for biomarkers in disease diagnosis; however, their practical utility in clinical settings necessitates further investigation.

Axin‐1, IL8, and MCP2 in AD; CXCL1, MIP1α, S100‐A12, EIF4EBP1, and CCL4 in PD; MIP1α, TNF‐β, IL1a, and CXCL11 in MS; M‐CSF1, CXCL11, TNFRSF9, IL10RA, IL12B, S100‐A12, CD6, and ANX, VEGFA, CXCL10 in DEP; and CXCL1, CDCP1, M‐CSF1, NKR2B4, and UE exhibited a strong intercorrelation. Our MR study did not reveal any evidence that establishes a causal link between systemic inflammatory factors and the onset of either PD or MS. Contrarily, it uncovered a causal connection between PD, MS, and specific systemic inflammatory regulatory factors. These findings imply that systemic inflammatory regulatory factors might be downstream consequences of PD and MS, or the result of co‐factors that induce inflammation and subsequently lead to PD and MS. The interplay between upstream factors, other systemic inflammatory regulatory factors, and PD, as well as MS can be further examined. This analysis is expected to yield additional evidence contributing to the etiological understanding of PD and MS. Regarding the associations between S100‐A12, TRANCE, and AD, as well as between inflammatory factors and ANX, IL12B, Casp‐8, and DEP, and between IL12B and UE, these are novel discoveries. The scarcity of relevant studies we encountered has impeded our understanding of these relationships. Consequently, these associations warrant further investigation through observational and experimental studies.

However, our research is also subject to certain limitations. First, owing to the scarcity of individual data, our GWAS data were exclusively derived from European populations. Additional investigations are necessary to determine if the results of the current study hold consistent for populations spanning different geographical areas. Second, while the MR‐Egger intercept did not suggest existing substantial horizontal pleiotropy, this possibility cannot be entirely ruled out. Finally, the adoption of a more lenient threshold for assessing the results may have resulted in the inclusion of some false‐positive findings.

In the later stage, in‐depth research can be conducted on the specific cellular and molecular mechanisms by which the changes in the concentrations of inflammatory factors trigger diseases. At the cellular level, it is necessary to study how these inflammatory factors affect the functions of neurons and the activities of glial cells. Moreover, clinical trials should be carried out to verify the effectiveness of these inflammatory factors in diagnosis and treatment. Track inflammatory factor levels during disease progression and treatment to improve diagnostic accuracy and refine treatment models.It is also important to further study the interaction networks among these molecules. For example, whether there is a mutual regulatory relationship between CD40L and IL17C and how they work synergistically in the pathogenesis of ANX and DEP. These studies will help us understand the pathogenesis of these diseases more comprehensively.

## 5 Conclusion

In conclusion, our study utilizing bidirectional MR points to a potential connection between a cluster of systemic inflammatory regulatory factors and the onset of neuropsychiatric disorders, or conversely, that the presence of certain neuropsychiatric conditions may affect the concentration levels of these factors. While additional studies are required to clarify the precise pathway by which systemic inflammatory regulatory factors impact neuropsychiatric disorders, the current study offers novel insights into the relationship between these factors and neuropsychiatric conditions. First, it offers new biomarkers for predicting mental disorders and may help prevent psychiatric disorders, also facilitating new therapeutic target development. Second, it aids in understanding pathophysiological connections like between PD and ANX. Third, it reveals common pathways among diseases and shows the immune system's role in neuropsychiatric disorders. For the future, in‐depth research on cellular and molecular mechanisms is needed, along with clinical trials to verify effectiveness and study interaction networks, which will comprehensively clarify disease pathogenesis.

## Author Contributions

**Hao Wang**: Conceptualization; methodology; writing—review and editing; writing—original draft; software; visualization; formal analysis; data curation; investigation. **Ziji Cheng**: Data curation; formal analysis. **Zixuan Xu**: Conceptualization; methodology; software. **Min Wang**: Conceptualization; methodology; software. **Xingyang Sun**: Data curation; investigation; validation. **Wen Liu**: Data curation; investigation; validation. **Jingtian Wang**: Data curation; investigation; validation. **Qian Yang**: Formal analysis; visualization. **Tuo Zhang**: Formal analysis; visualization. **Yanjun Du**: Resources; funding acquisition; supervision. **Xiaoming Zhang**: Supervision; funding acquisition; resources. **Jie Song**: Supervision; funding acquisition; resources; writing—review and editing; project administration.

## Ethics Statement

All data used in this study are publicly available and do not require ethical approval.

## Conflicts of Interest

The authors declare no conflicts of interest.

### Peer Review

The peer review history for this article is available at https://publons.com/publon/10.1002/brb3.70478

## Supporting information



Supplementary Materials.

Supplementary Materials.

Supplementary Materials.

Supplementary Materials.

## Data Availability

This study uses summary statistics from GWAS studies that are publicly available. Data on 91 of these systemic inflammatory modulators are available at https://figshare.com/articles/dataset/___/24958362?file = 43951080. Data on six neuropsychiatric disorders were obtained from Finnish databases, and the data for these six disorders were downloaded at AD: https://r10.finngen.fi/pheno/G6_AD_WIDE; PD: https://r10.finngen.fi/pheno/G6_PARKINSON; MS: https://r10.finngen.fi/pheno/G6_MS; ANX: https://r10.finngen.fi/pheno/KRA_PSY_ANXIETY_EXMORE; DEP: https://r10.finngen.fi/pheno/F5_DEPRESSIO; UE: https://r10.finngen.fi/pheno/G6_DISBROTHUNS?
