# Supplementary material for: Systemic Inflammatory Factors and Neuropsychiatric Disorders: A Bidirectional Mendelian Randomization Study
Source: Brain Behav. 2025 Apr 9;15(4):e70478. doi: 10.1002/brb3.70478 (PMC11979492; doi:10.1002/brb3.70478)
Supplement: Supplementary file 1 — Supplementary Materials. [file BRB3-15-e70478-s002.docx]

| S1. a detailed account of the outcomes of all these analyses. | | | | | | | | | | | |
| --- | --- | --- | --- | --- | --- | --- | --- | --- | --- | --- | --- |
| Outcome | Exposure | nSNP | MR_test | P_value | Pleiotropy_test | P for pleiotropy | Cochran's Q test | I2 | P for heterogeneity | beta | se |
| AD | NRTN | 26 | MR Egger | 0.016 | 0.011 | 0.188 | 30.391 | 0.21 | 0.172 | -0.184 | 0.071 |
|  | NRTN | 26 | Weighted median | 0.034 |  |  |  |  |  | -0.106 | 0.051 |
|  | NRTN | 26 | IVW | 0.009 |  |  | 32.713 | 0.24 | 0.138 | -0.104 | 0.039 |
|  | NRTN | 26 | Simple mode | 0.173 |  |  |  |  |  | -0.135 | 0.096 |
|  | NRTN | 26 | Weighted mode | 0.112 |  |  |  |  |  | -0.123 | 0.075 |
|  | S100-A12 | 24 | MR Egger | 0.513 | 0.004 | 0.629 | 23.976 | 0.08 | 0.348 | 0.053 | 0.079 |
|  | S100-A12 | 24 | Weighted median | 0.296 |  |  |  |  |  | 0.061 | 0.058 |
|  | S100-A12 | 24 | IVW | 0.026 |  |  | 24.238 | 0.05 | 0.391 | 0.086 | 0.039 |
|  | S100-A12 | 24 | Simple mode | 0.452 |  |  |  |  |  | 0.071 | 0.091 |
|  | S100-A12 | 24 | Weighted mode | 0.343 |  |  |  |  |  | 0.061 | 0.062 |
|  | IL-33 | 22 | MR Egger | 0.049 | -0.012 | 0.208 | 12.972 | 0 | 0.879 | 0.198 | 0.094 |
|  | IL-33 | 22 | Weighted median | 0.079 |  |  |  |  |  | 0.102 | 0.058 |
|  | IL-33 | 22 | IVW | 0.033 |  |  | 14.668 | 0 | 0.839 | 0.087 | 0.041 |
|  | IL-33 | 22 | Simple mode | 0.309 |  |  |  |  |  | 0.117 | 0.112 |
|  | IL-33 | 22 | Weighted mode | 0.206 |  |  |  |  |  | 0.123 | 0.094 |
|  | TRAIL | 41 | MR Egger | 0.043 | -0.006 | 0.326 | 56.449 | 0.31 | 0.135 | 0.088 | 0.042 |
|  | TRAIL | 41 | Weighted median | 0.011 |  |  |  |  |  | 0.083 | 0.032 |
|  | TRAIL | 41 | IVW | 0.034 |  |  | 57.881 | 0.31 | 0.133 | 0.056 | 0.026 |
|  | TRAIL | 41 | Simple mode | 0.137 |  |  |  |  |  | 0.085 | 0.056 |
|  | TRAIL | 41 | Weighted mode | 0.029 |  |  |  |  |  | 0.077 | 0.034 |
|  | TRANCE | 55 | MR Egger | 0.033 | -0.007 | 0.188 | 52.424 | 0 | 0.497 | 0.106 | 0.048 |
|  | TRANCE | 55 | Weighted median | 0.625 |  |  |  |  |  | 0.018 | 0.037 |
|  | TRANCE | 55 | IVW | 0.041 |  |  | 54.201 | 0 | 0.467 | 0.051 | 0.025 |
|  | TRANCE | 55 | Simple mode | 0.601 |  |  |  |  |  | 0.032 | 0.061 |
|  | TRANCE | 55 | Weighted mode | 0.623 |  |  |  |  |  | 0.021 | 0.041 |
| ANX | CD40L | 30 | MR Egger | 0.001 | 0.008 | 0.142 | 24.878 | 0 | 0.634 | -0.097 | 0.021 |
|  | CD40L | 30 | Weighted median | 0.001 |  |  |  |  |  | -0.084 | 0.018 |
|  | CD40L | 30 | IVW | 0.001 |  |  | 31.764 | 0.09 | 0.331 | -0.057 | 0.015 |
|  | CD40L | 30 | Simple mode | 0.917 |  |  |  |  |  | -0.006 | 0.057 |
|  | CD40L | 30 | Weighted mode | 0.001 |  |  |  |  |  | -0.083 | 0.017 |
|  | IL12B | 42 | MR Egger | 0.561 | 0.004 | 0.194 | 54.883 | 0.27 | 0.059 | 0.013 | 0.021 |
|  | IL12B | 42 | Weighted median | 0.221 |  |  |  |  |  | 0.021 | 0.017 |
|  | IL12B | 42 | IVW | 0.011 |  |  | 57.272 | 0.28 | 0.057 | 0.035 | 0.013 |
|  | IL12B | 42 | Simple mode | 0.241 |  |  |  |  |  | 0.039 | 0.033 |
|  | IL12B | 42 | Weighted mode | 0.059 |  |  |  |  |  | 0.028 | 0.014 |
|  | VEGFA | 41 | MR Egger | 0.007 | -0.005 | 0.119 | 47.091 | 0.17 | 0.175 | 0.063 | 0.022 |
|  | VEGFA | 41 | Weighted median | 0.002 |  |  |  |  |  | 0.056 | 0.018 |
|  | VEGFA | 41 | IVW | 0.015 |  |  | 50.166 | 0.2 | 0.131 | 0.036 | 0.015 |
|  | VEGFA | 41 | Simple mode | 0.638 |  |  |  |  |  | 0.024 | 0.051 |
|  | VEGFA | 41 | Weighted mode | 0.006 |  |  |  |  |  | 0.051 | 0.017 |
|  | Casp-8 | 23 | MR Egger | 0.067 | -0.004 | 0.426 | 22.998 | 0.09 | 0.344 | 0.094 | 0.049 |
|  | Casp-8 | 23 | Weighted median | 0.016 |  |  |  |  |  | 0.094 | 0.039 |
|  | Casp-8 | 23 | IVW | 0.018 |  |  | 23.72 | 0.07 | 0.362 | 0.061 | 0.026 |
|  | Casp-8 | 23 | Simple mode | 0.137 |  |  |  |  |  | 0.105 | 0.068 |
|  | Casp-8 | 23 | Weighted mode | 0.075 |  |  |  |  |  | 0.105 | 0.056 |
|  | TNFRSF9 | 35 | MR Egger | 0.218 | -0.001 | 0.827 | 24.379 | 0 | 0.861 | 0.051 | 0.041 |
|  | TNFRSF9 | 35 | Weighted median | 0.081 |  |  |  |  |  | 0.047 | 0.027 |
|  | TNFRSF9 | 35 | IVW | 0.022 |  |  | 24.427 | 0 | 0.887 | 0.043 | 0.019 |
|  | TNFRSF9 | 35 | Simple mode | 0.335 |  |  |  |  |  | 0.051 | 0.051 |
|  | TNFRSF9 | 35 | Weighted mode | 0.275 |  |  |  |  |  | 0.051 | 0.046 |
|  | IL18R1 | 50 | MR Egger | 0.079 | -0.001 | 0.823 | 69.165 | 0.31 | 0.054 | 0.027 | 0.015 |
|  | IL18R1 | 50 | Weighted median | 0.037 |  |  |  |  |  | 0.029 | 0.014 |
|  | IL18R1 | 50 | IVW | 0.023 |  |  | 69.237 | 0.29 | 0.053 | 0.024 | 0.011 |
|  | IL18R1 | 50 | Simple mode | 0.437 |  |  |  |  |  | 0.024 | 0.031 |
|  | IL18R1 | 50 | Weighted mode | 0.021 |  |  |  |  |  | 0.024 | 0.011 |
|  | OPG | 32 | MR Egger | 0.297 | 0.001 | 0.987 | 23.585 | 0 | 0.791 | -0.045 | 0.043 |
|  | OPG | 32 | Weighted median | 0.495 |  |  |  |  |  | -0.021 | 0.029 |
|  | OPG | 32 | IVW | 0.024 |  |  | 23.586 | 0 | 0.827 | -0.046 | 0.021 |
|  | OPG | 32 | Simple mode | 0.631 |  |  |  |  |  | -0.027 | 0.055 |
|  | OPG | 32 | Weighted mode | 0.537 |  |  |  |  |  | -0.023 | 0.038 |
|  | IL-10RA | 19 | MR Egger | 0.179 | -0.001 | 0.772 | 14.164 | 0 | 0.655 | 0.061 | 0.043 |
|  | IL-10RA | 19 | Weighted median | 0.035 |  |  |  |  |  | 0.075 | 0.035 |
|  | IL-10RA | 19 | IVW | 0.037 |  |  | 14.251 | 0 | 0.713 | 0.051 | 0.024 |
|  | IL-10RA | 19 | Simple mode | 0.152 |  |  |  |  |  | 0.089 | 0.059 |
|  | IL-10RA | 19 | Weighted mode | 0.065 |  |  |  |  |  | 0.083 | 0.042 |
| DEP | VEGF-A | 41 | MR Egger | 0.033 | -0.002 | 0.468 | 47.061 | 0.17 | 0.176 | 0.046 | 0.021 |
|  | VEGF-A | 41 | Weighted median | 0.005 |  |  |  |  |  | 0.045 | 0.016 |
|  | VEGF-A | 41 | IVW | 0.011 |  |  | 47.706 | 0.16 | 0.188 | 0.034 | 0.014 |
|  | VEGF-A | 41 | Simple mode | 0.922 |  |  |  |  |  | -0.005 | 0.047 |
|  | VEGF-A | 41 | Weighted mode | 0.004 |  |  |  |  |  | 0.045 | 0.015 |
|  | CD40L | 30 | MR Egger | 0.002 | 0.006 | 0.056 | 20.201 | 0 | 0.857 | -0.066 | 0.021 |
|  | CD40L | 30 | Weighted median | 0.003 |  |  |  |  |  | -0.052 | 0.017 |
|  | CD40L | 30 | IVW | 0.017 |  |  | 25.695 | 0 | 0.642 | -0.032 | 0.013 |
|  | CD40L | 30 | Simple mode | 0.725 |  |  |  |  |  | -0.016 | 0.046 |
|  | CD40L | 30 | Weighted mode | 0.004 |  |  |  |  |  | -0.051 | 0.017 |
|  | ADA | 30 | MR Egger | 0.283 | -0.003 | 0.375 | 22.801 | 0 | 0.743 | -0.019 | 0.017 |
|  | ADA | 30 | Weighted median | 0.027 |  |  |  |  |  | -0.035 | 0.016 |
|  | ADA | 30 | IVW | 0.026 |  |  | 23.613 | 0 | 0.748 | -0.029 | 0.013 |
|  | ADA | 30 | Simple mode | 0.481 |  |  |  |  |  | -0.032 | 0.045 |
|  | ADA | 30 | Weighted mode | 0.034 |  |  |  |  |  | -0.034 | 0.015 |
|  | IL12B | 42 | MR Egger | 0.467 | 0.002 | 0.521 | 63.488 | 0.37 | 0.11 | 0.016 | 0.022 |
|  | IL12B | 42 | Weighted median | 0.071 |  |  |  |  |  | 0.028 | 0.015 |
|  | IL12B | 42 | IVW | 0.044 |  |  | 64.157 | 0.36 | 0.112 | 0.027 | 0.013 |
|  | IL12B | 42 | Simple mode | 0.266 |  |  |  |  |  | 0.035 | 0.031 |
|  | IL12B | 42 | Weighted mode | 0.031 |  |  |  |  |  | 0.031 | 0.013 |
|  | IL18R1 | 50 | MR Egger | 0.045 | -0.003 | 0.375 | 69.013 | 0.3 | 0.055 | 0.029 | 0.014 |
|  | IL18R1 | 50 | Weighted median | 0.087 |  |  |  |  |  | 0.021 | 0.012 |
|  | IL18R1 | 50 | IVW | 0.046 |  |  | 70.165 | 0.3 | 0.056 | 0.021 | 0.011 |
|  | IL18R1 | 50 | Simple mode | 0.566 |  |  |  |  |  | 0.017 | 0.029 |
|  | IL18R1 | 50 | Weighted mode | 0.047 |  |  |  |  |  | 0.019 | 0.011 |
|  | Casp-8 | 23 | MR Egger | 0.071 | -0.005 | 0.321 | 19.905 | 0 | 0.527 | 0.083 | 0.043 |
|  | Casp-8 | 23 | Weighted median | 0.028 |  |  |  |  |  | 0.072 | 0.033 |
|  | Casp-8 | 23 | IVW | 0.048 |  |  | 20.939 | 0 | 0.525 | 0.046 | 0.023 |
|  | Casp-8 | 23 | Simple mode | 0.089 |  |  |  |  |  | 0.096 | 0.054 |
|  | Casp-8 | 23 | Weighted mode | 0.064 |  |  |  |  |  | 0.088 | 0.045 |
| UE | IL12B | 30 | MR Egger | 0.092 | -0.001 | 0.969 | 6.992 | 0 | 1 | 0.188 | 0.215 |
|  | IL12B | 30 | Weighted median | 0.061 |  |  |  |  |  | 0.121 | 0.171 |
|  | IL12B | 30 | IVW | 0.032 |  |  | 6.994 | 0 | 1 | 0.281 | 0.201 |
|  | IL12B | 30 | Simple mode | 0.026 |  |  |  |  |  | 0.129 | 0.129 |
|  | IL12B | 30 | Weighted mode | 0.074 |  |  |  |  |  | 0.201 | 0.212 |
