# Supplementary material for: Systemic Inflammatory Factors and Neuropsychiatric Disorders: A Bidirectional Mendelian Randomization Study
Source: Brain Behav. 2025 Apr 9;15(4):e70478. doi: 10.1002/brb3.70478 (PMC11979492; doi:10.1002/brb3.70478)

Outcome	Exposure	nSNP	MR_test	P_value	Pleiotropy_test	P.for.pleiotropy	Cochran.s.Q.test	I2	P.for.heterogeneity
AD	NRTN	26	MR Egger	0.016	0.011	0.188	30.391	0.21	0.172
	NRTN	26	Weighted median	0.034					
	NRTN	26	Inverse variance weighted	0.009			32.713	0.24	0.138
	NRTN	26	Simple mode	0.173					
	NRTN	26	Weighted mode	0.112					
	S100−A12	24	MR Egger	0.513	0.004	0.629	23.976	0.08	0.348
	S100−A12	24	Weighted median	0.296					
	S100−A12	24	Inverse variance weighted	0.026			24.238	0.05	0.391
	S100−A12	24	Simple mode	0.452					
	S100−A12	24	Weighted mode	0.343					
	IL−33	22	MR Egger	0.049	−0.012	0.208	12.972	0	0.879
	IL−33	22	Weighted median	0.079					
	IL−33	22	Inverse variance weighted	0.033			14.668	0	0.839
	IL−33	22	Simple mode	0.309					
	IL−33	22	Weighted mode	0.206					
	TRAIL	41	MR Egger	0.043	−0.006	0.326	56.449	0.31	0.135
	TRAIL	41	Weighted median	0.011					
	TRAIL	41	Inverse variance weighted	0.034			57.881	0.31	0.133
	TRAIL	41	Simple mode	0.137					
	TRAIL	41	Weighted mode	0.029					
	TRANCE	55	MR Egger	0.033	−0.007	0.188	52.424	0	0.497
	TRANCE	55	Weighted median	0.625					
	TRANCE	55	Inverse variance weighted	0.041			54.201	0	0.467
	TRANCE	55	Simple mode	0.601					
	TRANCE	55	Weighted mode	0.623					
ANX	CD40L	30	MR Egger	0.001	0.008	0.142	24.878	0	0.634
	CD40L	30	Weighted median	0.001					
	CD40L	30	Inverse variance weighted	0.001			31.764	0.09	0.331
	CD40L	30	Simple mode	0.917					
	CD40L	30	Weighted mode	0.001					
	IL12B	42	MR Egger	0.561	0.004	0.194	54.883	0.27	0.059
	IL12B	42	Weighted median	0.221					
	IL12B	42	Inverse variance weighted	0.011			57.272	0.28	0.057
	IL12B	42	Simple mode	0.241					
	IL12B	42	Weighted mode	0.059					
	VEGFA	41	MR Egger	0.007	−0.005	0.119	47.091	0.17	0.175
	VEGFA	41	Weighted median	0.002					
	VEGFA	41	Inverse variance weighted	0.015			50.166	0.2	0.131
	VEGFA	41	Simple mode	0.638					
	VEGFA	41	Weighted mode	0.006					
	Casp−8	23	MR Egger	0.067	−0.004	0.426	22.998	0.09	0.344
	Casp−8	23	Weighted median	0.016					
	Casp−8	23	Inverse variance weighted	0.018			23.72	0.07	0.362
	Casp−8	23	Simple mode	0.137					
	Casp−8	23	Weighted mode	0.075					
	TNFRSF9	35	MR Egger	0.218	−0.001	0.827	24.379	0	0.861
	TNFRSF9	35	Weighted median	0.081					
	TNFRSF9	35	Inverse variance weighted	0.022			24.427	0	0.887
	TNFRSF9	35	Simple mode	0.335					
	TNFRSF9	35	Weighted mode	0.275					
	IL18R1	50	MR Egger	0.079	−0.001	0.823	69.165	0.31	0.054
	IL18R1	50	Weighted median	0.037					
	IL18R1	50	Inverse variance weighted	0.023			69.237	0.29	0.053
	IL18R1	50	Simple mode	0.437					
	IL18R1	50	Weighted mode	0.021					
	OPG	32	MR Egger	0.297	0.001	0.987	23.585	0	0.791
	OPG	32	Weighted median	0.495					
	OPG	32	Inverse variance weighted	0.024			23.586	0	0.827
	OPG	32	Simple mode	0.631					
	OPG	32	Weighted mode	0.537					
	IL−10RA	19	MR Egger	0.179	−0.001	0.772	14.164	0	0.655
	IL−10RA	19	Weighted median	0.035					
	IL−10RA	19	Inverse variance weighted	0.037			14.251	0	0.713
	IL−10RA	19	Simple mode	0.152					
	IL−10RA	19	Weighted mode	0.065					
DEP	VEGF−A	41	MR Egger	0.033	−0.002	0.468	47.061	0.17	0.176
	VEGF−A	41	Weighted median	0.005					
	VEGF−A	41	Inverse variance weighted	0.011			47.706	0.16	0.188
	VEGF−A	41	Simple mode	0.922					
	VEGF−A	41	Weighted mode	0.004					
	CD40L	30	MR Egger	0.002	0.006	0.056	20.201	0	0.857
	CD40L	30	Weighted median	0.003					
	CD40L	30	Inverse variance weighted	0.017			25.695	0	0.642
	CD40L	30	Simple mode	0.725					
	CD40L	30	Weighted mode	0.004					
	ADA	30	MR Egger	0.283	−0.003	0.375	22.801	0	0.743
	ADA	30	Weighted median	0.027					
	ADA	30	Inverse variance weighted	0.026			23.613	0	0.748
	ADA	30	Simple mode	0.481					
	ADA	30	Weighted mode	0.034					
	IL12B	42	MR Egger	0.467	0.002	0.521	63.488	0.37	0.11
	IL12B	42	Weighted median	0.071					
	IL12B	42	Inverse variance weighted	0.044			64.157	0.36	0.112
	IL12B	42	Simple mode	0.266					
	IL12B	42	Weighted mode	0.031					
	IL18R1	50	MR Egger	0.045	−0.003	0.375	69.013	0.3	0.055
	IL18R1	50	Weighted median	0.087					
	IL18R1	50	Inverse variance weighted	0.046			70.165	0.3	0.056
	IL18R1	50	Simple mode	0.566					
	IL18R1	50	Weighted mode	0.047					
	Casp−8	23	MR Egger	0.071	−0.005	0.321	19.905	0	0.527
	Casp−8	23	Weighted median	0.028					
	Casp−8	23	Inverse variance weighted	0.048			20.939	0	0.525
	Casp−8	23	Simple mode	0.089					
	Casp−8	23	Weighted mode	0.064					
UE	IL12B	30	MR Egger	0.092	−0.001	0.969	6.992	0	1
	IL12B	30	Weighted median	0.061					
	IL12B	30	Inverse variance weighted	0.032			6.994	0	1
	IL12B	30	Simple mode	0.026					
	IL12B	30	Weighted mode	0.074					

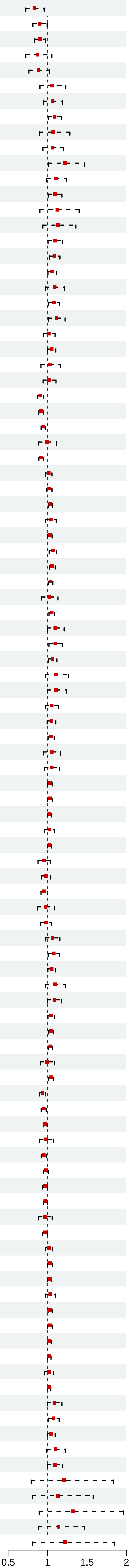

Supplement: Supplementary file 2 — Supplementary Materials. [file BRB3-15-e70478-s004.pdf]
