# Supplementary material for: Systemic Inflammatory Factors and Neuropsychiatric Disorders: A Bidirectional Mendelian Randomization Study
Source: Brain Behav. 2025 Apr 9;15(4):e70478. doi: 10.1002/brb3.70478 (PMC11979492; doi:10.1002/brb3.70478)
Supplement: Supplementary file 3 — Supplementary Materials. [file BRB3-15-e70478-s003.docx]

| S3：Results of the analysis of the impact of the onset of neuropsychiatric disorders on the levels of systemic inflammatory factors | | | | | | | | | | | |
| --- | --- | --- | --- | --- | --- | --- | --- | --- | --- | --- | --- |
| Exposure | Outcome | nSNP | MR_test | P_value | Pleiotropy_test | P for pleiotropy | Cochran's Q test | I2 | P for heterogeneity | beta | se |
| AD | CDCP 1 | 44 | MR Egger | 0.042 | 0.002 | 0.751 | 45.021 | 0.07 | 0.347 | -0.099 | 0.047 |
|  |  | 44 | Weighted median | 0.018 |  |  |  |  |  | -0.102 | 0.043 |
|  |  | 44 | IVW | 0.002 |  |  | 45.131 | 0.05 | 0.383 | -0.087 | 0.028 |
|  |  | 44 | Simple mode | 0.053 |  |  |  |  |  | -0.151 | 0.075 |
|  |  | 44 | Weighted mode | 0.033 |  |  |  |  |  | -0.111 | 0.051 |
|  | Axin-1 | 25 | MR Egger | 0.127 | 0.001 | 0.939 | 36.954 | 0.38 | 0.053 | 0.138 | 0.087 |
|  |  | 25 | Weighted median | 0.023 |  |  |  |  |  | 0.145 | 0.064 |
|  |  | 25 | IVW | 0.008 |  |  | 36.964 | 0.35 | 0.054 | 0.143 | 0.054 |
|  |  | 25 | Simple mode | 0.048 |  |  |  |  |  | 0.251 | 0.121 |
|  |  | 25 | Weighted mode | 0.068 |  |  |  |  |  | 0.183 | 0.095 |
|  | IL8 | 38 | MR Egger | 0.024 | -0.004 | 0.442 | 24.314 | 0 | 0.931 | 0.086 | 0.036 |
|  |  | 38 | Weighted median | 0.032 |  |  |  |  |  | 0.081 | 0.038 |
|  |  | 38 | IVW | 0.013 |  |  | 24.92 | 0 | 0.935 | 0.067 | 0.027 |
|  |  | 38 | Simple mode | 0.136 |  |  |  |  |  | 0.097 | 0.064 |
|  |  | 38 | Weighted mode | 0.031 |  |  |  |  |  | 0.094 | 0.042 |
|  | MCP2 | 9 | MR Egger | 0.071 | -0.007 | 0.56 | 5.222 | 0 | 0.633 | 0.052 | 0.024 |
|  |  | 9 | Weighted median | 0.022 |  |  |  |  |  | 0.047 | 0.021 |
|  |  | 9 | IVW | 0.026 |  |  | 5.595 | 0 | 0.692 | 0.043 | 0.019 |
|  |  | 9 | Simple mode | 0.321 |  |  |  |  |  | 0.083 | 0.078 |
|  |  | 9 | Weighted mode | 0.055 |  |  |  |  |  | 0.046 | 0.021 |
|  | IL20 | 37 | MR Egger | 0.018 | 0.011 | 0.191 | 28.662 | 0 | 0.767 | -0.064 | 0.026 |
|  |  | 37 | Weighted median | 0.103 |  |  |  |  |  | -0.051 | 0.031 |
|  |  | 37 | IVW | 0.033 |  |  | 30.443 | 0 | 0.731 | -0.042 | 0.021 |
|  |  | 37 | Simple mode | 0.999 |  |  |  |  |  | -0.001 | 0.061 |
|  |  | 37 | Weighted mode | 0.088 |  |  |  |  |  | -0.047 | 0.027 |
| PD | SULT1A1 | 47 | MR Egger | 0.153 | -0.001 | 0.929 | 52.412 | 0.14 | 0.209 | -0.101 | 0.071 |
|  |  | 47 | Weighted median | 0.146 |  |  |  |  |  | -0.088 | 0.061 |
|  |  | 47 | IVW | 0.011 |  |  | 52.421 | 0.12 | 0.239 | -0.106 | 0.042 |
|  |  | 47 | Simple mode | 0.249 |  |  |  |  |  | -0.138 | 0.118 |
|  |  | 47 | Weighted mode | 0.345 |  |  |  |  |  | -0.081 | 0.085 |
|  | IL-15RA | 31 | MR Egger | 0.399 | -0.011 | 0.382 | 26.579 | 0 | 0.594 | -0.051 | 0.061 |
|  |  | 31 | Weighted median | 0.061 |  |  |  |  |  | -0.102 | 0.054 |
|  |  | 31 | IVW | 0.014 |  |  | 27.367 | 0 | 0.604 | -0.093 | 0.038 |
|  |  | 31 | Simple mode | 0.555 |  |  |  |  |  | -0.065 | 0.109 |
|  |  | 31 | Weighted mode | 0.042 |  |  |  |  |  | -0.098 | 0.046 |
|  | CXCL1 | 37 | MR Egger | 0.071 | -0.003 | 0.786 | 40.912 | 0.14 | 0.227 | 0.135 | 0.072 |
|  |  | 37 | Weighted median | 0.055 |  |  |  |  |  | 0.138 | 0.072 |
|  |  | 37 | IVW | 0.015 |  |  | 40.999 | 0.12 | 0.261 | 0.121 | 0.051 |
|  |  | 37 | Simple mode | 0.132 |  |  |  |  |  | 0.158 | 0.102 |
|  |  | 37 | Weighted mode | 0.029 |  |  |  |  |  | 0.145 | 0.064 |
|  | MIP1a | 40 | MR Egger | 0.014 | -0.014 | 0.143 | 35.925 | 0 | 0.566 | 0.141 | 0.055 |
|  |  | 40 | Weighted median | 0.024 |  |  |  |  |  | 0.129 | 0.057 |
|  |  | 40 | IVW | 0.031 |  |  | 38.165 | 0 | 0.508 | 0.084 | 0.039 |
|  |  | 40 | Simple mode | 0.068 |  |  |  |  |  | 0.221 | 0.118 |
|  |  | 40 | Weighted mode | 0.006 |  |  |  |  |  | 0.144 | 0.049 |
|  | S100-A12 | 45 | MR Egger | 0.056 | 0.009 | 0.364 | 42.104 | 0 | 0.511 | 0.051 | 0.067 |
|  |  | 45 | Weighted median | 0.012 |  |  |  |  |  | 0.051 | 0.077 |
|  |  | 45 | IVW | 0.032 |  |  | 42.945 | 0 | 0.517 | 0.096 | 0.045 |
|  |  | 45 | Simple mode | 0.063 |  |  |  |  |  | 0.081 | 0.138 |
|  |  | 45 | Weighted mode | 0.074 |  |  |  |  |  | 0.047 | 0.111 |
|  | EIF4EBP1 | 36 | MR Egger | 0.232 | 0.003 | 0.745 | 31.191 | 0 | 0.606 | 0.101 | 0.051 |
|  |  | 36 | Weighted median | 0.282 |  |  |  |  |  | 0.085 | 0.071 |
|  |  | 36 | IVW | 0.043 |  |  | 31.299 | 0 | 0.647 | 0.085 | 0.079 |
|  |  | 36 | Simple mode | 0.042 |  |  |  |  |  | -0.026 | 0.128 |
|  |  | 36 | Weighted mode | 0.093 |  |  |  |  |  | 0.025 | 0.095 |
|  | PD-L1 | 37 | MR Egger | 0.106 | 0.006 | 0.588 | 33.915 | 0 | 0.521 | -0.161 | 0.096 |
|  |  | 37 | Weighted median | 0.062 |  |  |  |  |  | -0.168 | 0.091 |
|  |  | 37 | IVW | 0.047 |  |  | 34.213 | 0 | 0.554 | -0.118 | 0.059 |
|  |  | 37 | Simple mode | 0.067 |  |  |  |  |  | -0.324 | 0.171 |
|  |  | 37 | Weighted mode | 0.118 |  |  |  |  |  | -0.193 | 0.121 |
|  | CCL4 | 41 | MR Egger | 0.162 | 0.001 | 0.948 | 38.932 | 0 | 0.473 | 0.059 | 0.042 |
|  |  | 41 | Weighted median | 0.139 |  |  |  |  |  | 0.067 | 0.045 |
|  |  | 41 | IVW | 0.048 |  |  | 38.936 | 0 | 0.518 | 0.061 | 0.031 |
|  |  | 41 | Simple mode | 0.031 |  |  |  |  |  | 0.091 | 0.093 |
|  |  | 41 | Weighted mode | 0.171 |  |  |  |  |  | 0.054 | 0.038 |
| MS | MIP-1a | 40 | MR Egger | 0.204 | 0.021 | 0.141 | 40.758 | 0.07 | 0.351 | 0.101 | 0.078 |
|  |  | 40 | Weighted median | 0.193 |  |  |  |  |  | 0.103 | 0.079 |
|  |  | 40 | IVW | 0.001 |  |  | 43.202 | 0.1 | 0.296 | 0.182 | 0.057 |
|  |  | 40 | Simple mode | 0.014 |  |  |  |  |  | 0.373 | 0.145 |
|  |  | 40 | Weighted mode | 0.031 |  |  |  |  |  | 0.146 | 0.065 |
|  | TNF-beta | 8 | MR Egger | 0.609 | 0.102 | 0.199 | 10.787 | 0.44 | 0.095 | -0.249 | 0.262 |
|  |  | 8 | Weighted median | 0.001 |  |  |  |  |  | 0.554 | 0.166 |
|  |  | 8 | IVW | 0.004 |  |  | 14.529 | 0.52 | 0.053 | 0.391 | 0.137 |
|  |  | 8 | Simple mode | 0.048 |  |  |  |  |  | 0.521 | 0.201 |
|  |  | 8 | Weighted mode | 0.826 |  |  |  |  |  | 0.047 | 0.206 |
|  | CD40L | 37 | MR Egger | 0.032 | 0.011 | 0.408 | 36.274 | 0.04 | 0.409 | -0.168 | 0.075 |
|  |  | 37 | Weighted median | 0.001 |  |  |  |  |  | -0.227 | 0.069 |
|  |  | 37 | IVW | 0.023 |  |  | 37.001 | 0.03 | 0.423 | -0.126 | 0.056 |
|  |  | 37 | Simple mode | 0.203 |  |  |  |  |  | -0.266 | 0.205 |
|  |  | 37 | Weighted mode | 0.002 |  |  |  |  |  | -0.224 | 0.066 |
|  | IL1a | 34 | MR Egger | 0.219 | 0.005 | 0.811 | 42.035 | 0.24 | 0.111 | 0.155 | 0.124 |
|  |  | 34 | Weighted median | 0.387 |  |  |  |  |  | 0.108 | 0.125 |
|  |  | 34 | IVW | 0.033 |  |  | 42.111 | 0.22 | 0.133 | 0.177 | 0.083 |
|  |  | 34 | Simple mode | 0.859 |  |  |  |  |  | -0.036 | 0.204 |
|  |  | 34 | Weighted mode | 0.613 |  |  |  |  |  | 0.078 | 0.152 |
|  | Artemin | 39 | MR Egger | 0.041 | 0.015 | 0.347 | 27.601 | 0 | 0.869 | -0.239 | 0.113 |
|  |  | 39 | Weighted median | 0.454 |  |  |  |  |  | -0.082 | 0.111 |
|  |  | 39 | IVW | 0.034 |  |  | 28.508 | 0 | 0.868 | -0.157 | 0.074 |
|  |  | 39 | Simple mode | 0.366 |  |  |  |  |  | -0.191 | 0.207 |
|  |  | 39 | Weighted mode | 0.588 |  |  |  |  |  | -0.074 | 0.135 |
|  | CXC11 | 51 | MR Egger | 0.011 | -0.026 | 0.092 | 67.854 | 0.28 | 0.058 | 0.287 | 0.108 |
|  |  | 51 | Weighted median | 0.064 |  |  |  |  |  | 0.172 | 0.093 |
|  |  | 51 | IVW | 0.04 |  |  | 71.944 | 0.31 | 0.053 | 0.144 | 0.071 |
|  |  | 51 | Simple mode | 0.847 |  |  |  |  |  | -0.039 | 0.201 |
|  |  | 51 | Weighted mode | 0.337 |  |  |  |  |  | 0.099 | 0.102 |
| ANX | CD40L | 37 | MR Egger | 0.001 | 0.007 | 0.058 | 30.771 | 0 | 0.673 | -0.079 | 0.021 |
|  |  | 37 | Weighted median | 0.001 |  |  |  |  |  | -0.084 | 0.019 |
|  |  | 37 | IVW | 0.001 |  |  | 35.436 | 0 | 0.495 | -0.051 | 0.015 |
|  |  | 37 | Simple mode | 0.462 |  |  |  |  |  | -0.042 | 0.056 |
|  |  | 37 | Weighted mode | 0.001 |  |  |  |  |  | -0.077 | 0.019 |
|  | M-CSF1 | 38 | MR Egger | 0.202 | 0.003 | 0.516 | 46.648 | 0 | 0.111 | 0.041 | 0.031 |
|  |  | 38 | Weighted median | 0.147 |  |  |  |  |  | 0.041 | 0.028 |
|  |  | 38 | IVW | 0.006 |  |  | 47.206 | 0 | 0.121 | 0.055 | 0.021 |
|  |  | 38 | Simple mode | 0.043 |  |  |  |  |  | 0.039 | 0.051 |
|  |  | 38 | Weighted mode | 0.249 |  |  |  |  |  | 0.039 | 0.034 |
|  | CXL11 | 51 | MR Egger | 0.031 | -0.002 | 0.539 | 42.221 | 0 | 0.743 | 0.053 | 0.024 |
|  |  | 51 | Weighted median | 0.056 |  |  |  |  |  | 0.047 | 0.025 |
|  |  | 51 | IVW | 0.007 |  |  | 42.604 | 0 | 0.762 | 0.042 | 0.015 |
|  |  | 51 | Simple mode | 0.186 |  |  |  |  |  | 0.065 | 0.048 |
|  |  | 51 | Weighted mode | 0.057 |  |  |  |  |  | 0.057 | 0.029 |
|  | OPG | 38 | MR Egger | 0.437 | -0.005 | 0.179 | 19.754 | 0 | 0.987 | -0.021 | 0.027 |
|  |  | 38 | Weighted median | 0.492 |  |  |  |  |  | -0.021 | 0.029 |
|  |  | 38 | IVW | 0.007 |  |  | 21.634 | 0 | 0.979 | -0.049 | 0.018 |
|  |  | 38 | Simple mode | 0.401 |  |  |  |  |  | -0.038 | 0.044 |
|  |  | 38 | Weighted mode | 0.438 |  |  |  |  |  | -0.021 | 0.026 |
|  | IL17C | 38 | MR Egger | 0.793 | -0.007 | 0.065 | 35.596 | 0 | 0.488 | -0.007 | 0.025 |
|  |  | 38 | Weighted median | 0.193 |  |  |  |  |  | -0.033 | 0.026 |
|  |  | 38 | IVW | 0.011 |  |  | 39.904 | 0.07 | 0.342 | -0.045 | 0.018 |
|  |  | 38 | Simple mode | 0.241 |  |  |  |  |  | -0.051 | 0.043 |
|  |  | 38 | Weighted mode | 0.298 |  |  |  |  |  | -0.028 | 0.026 |
|  | TNFRSF9 | 43 | MR Egger | 0.109 | -0.001 | 0.818 | 33.028 | 0 | 0.808 | 0.053 | 0.032 |
|  |  | 43 | Weighted median | 0.055 |  |  |  |  |  | 0.052 | 0.027 |
|  |  | 43 | IVW | 0.011 |  |  | 33.082 | 0 | 0.836 | 0.047 | 0.018 |
|  |  | 43 | Simple mode | 0.271 |  |  |  |  |  | 0.059 | 0.053 |
|  |  | 43 | Weighted mode | 0.171 |  |  |  |  |  | 0.067 | 0.048 |
|  | CCL19 | 41 | MR Egger | 0.418 | -0.004 | 0.348 | 36.338 | 0 | 0.592 | -0.022 | 0.027 |
|  |  | 41 | Weighted median | 0.091 |  |  |  |  |  | -0.045 | 0.026 |
|  |  | 41 | IVW | 0.017 |  |  | 37.241 | 0 | 0.595 | -0.042 | 0.018 |
|  |  | 41 | Simple mode | 0.994 |  |  |  |  |  | 0.001 | 0.049 |
|  |  | 41 | Weighted mode | 0.109 |  |  |  |  |  | -0.047 | 0.029 |
|  | IL10RA | 43 | MR Egger | 0.076 | 0.001 | 0.996 | 43.772 | 0.06 | 0.355 | 0.034 | 0.019 |
|  |  | 43 | Weighted median | 0.645 |  |  |  |  |  | 0.011 | 0.023 |
|  |  | 43 | IVW | 0.017 |  |  | 43.772 | 0.04 | 0.396 | 0.034 | 0.014 |
|  |  | 43 | Simple mode | 0.381 |  |  |  |  |  | 0.033 | 0.037 |
|  |  | 43 | Weighted mode | 0.292 |  |  |  |  |  | 0.025 | 0.023 |
|  | IL12B | 20 | MR Egger | 0.364 | 0.002 | 0.807 | 30.631 | 0.41 | 0.052 | 0.047 | 0.051 |
|  |  | 20 | Weighted median | 0.103 |  |  |  |  |  | 0.046 | 0.028 |
|  |  | 20 | IVW | 0.021 |  |  | 30.736 | 0.38 | 0.053 | 0.058 | 0.025 |
|  |  | 20 | Simple mode | 0.465 |  |  |  |  |  | 0.041 | 0.055 |
|  |  | 20 | Weighted mode | 0.105 |  |  |  |  |  | 0.048 | 0.028 |
|  | IL24 | 29 | MR Egger | 0.503 | -0.008 | 0.181 | 32.611 | 0.17 | 0.211 | -0.021 | 0.031 |
|  |  | 29 | Weighted median | 0.552 |  |  |  |  |  | -0.019 | 0.032 |
|  |  | 29 | IVW | 0.025 |  |  | 34.896 | 0.2 | 0.173 | -0.051 | 0.023 |
|  |  | 29 | Simple mode | 0.291 |  |  |  |  |  | -0.068 | 0.063 |
|  |  | 29 | Weighted mode | 0.752 |  |  |  |  |  | -0.012 | 0.037 |
|  | S100-A12 | 45 | MR Egger | 0.189 | 0.001 | 0.901 | 58.794 | 0.27 | 0.055 | 0.038 | 0.028 |
|  |  | 45 | Weighted median | 0.585 |  |  |  |  |  | 0.014 | 0.026 |
|  |  | 45 | IVW | 0.031 |  |  | 58.815 | 0.25 | 0.067 | 0.041 | 0.019 |
|  |  | 45 | Simple mode | 0.409 |  |  |  |  |  | 0.041 | 0.049 |
|  |  | 45 | Weighted mode | 0.734 |  |  |  |  |  | 0.013 | 0.038 |
|  | CD6 | 5 | MR Egger | 0.176 | -0.002 | 0.738 | 2.654 | 0 | 0.448 | 0.036 | 0.021 |
|  |  | 5 | Weighted median | 0.042 |  |  |  |  |  | 0.032 | 0.016 |
|  |  | 5 | IVW | 0.047 |  |  | 2.788 | 0 | 0.594 | 0.031 | 0.016 |
|  |  | 5 | Simple mode | 0.677 |  |  |  |  |  | 0.019 | 0.042 |
|  |  | 5 | Weighted mode | 0.137 |  |  |  |  |  | 0.032 | 0.017 |
| DEP | VEGFA | 42 | MR Egger | 0.091 | 0.001 | 0.745 | 45.192 | 0.11 | 0.264 | 0.031 | 0.018 |
|  |  | 42 | Weighted median | 0.004 |  |  |  |  |  | 0.046 | 0.016 |
|  |  | 42 | IVW | 0.006 |  |  | 45.313 | 0.1 | 0.297 | 0.035 | 0.013 |
|  |  | 42 | Simple mode | 0.513 |  |  |  |  |  | 0.026 | 0.039 |
|  |  | 42 | Weighted mode | 0.006 |  |  |  |  |  | 0.042 | 0.014 |
|  | IL17C | 38 | MR Egger | 0.224 | -0.003 | 0.392 | 39.806 | 0.1 | 0.305 | -0.031 | 0.025 |
|  |  | 38 | Weighted median | 0.025 |  |  |  |  |  | -0.056 | 0.025 |
|  |  | 38 | IVW | 0.006 |  |  | 40.635 | 0.09 | 0.313 | -0.046 | 0.017 |
|  |  | 38 | Simple mode | 0.975 |  |  |  |  |  | 0.001 | 0.045 |
|  |  | 38 | Weighted mode | 0.045 |  |  |  |  |  | -0.057 | 0.028 |
|  | CXCL10 | 45 | MR Egger | 0.065 | 0.001 | 0.605 | 38.712 | 0 | 0.658 | 0.027 | 0.014 |
|  |  | 45 | Weighted median | 0.428 |  |  |  |  |  | 0.013 | 0.016 |
|  |  | 45 | IVW | 0.008 |  |  | 38.983 | 0 | 0.686 | 0.031 | 0.012 |
|  |  | 45 | Simple mode | 0.272 |  |  |  |  |  | 0.041 | 0.037 |
|  |  | 45 | Weighted mode | 0.392 |  |  |  |  |  | 0.014 | 0.016 |
|  | ADA | 5 | MR Egger | 0.205 | -0.001 | 0.834 | 2.085 | 0 | 0.555 | -0.031 | 0.019 |
|  |  | 5 | Weighted median | 0.029 |  |  |  |  |  | -0.035 | 0.016 |
|  |  | 5 | IVW | 0.033 |  |  | 2.137 | 0 | 0.711 | -0.034 | 0.016 |
|  |  | 5 | Simple mode | 0.809 |  |  |  |  |  | 0.011 | 0.041 |
|  |  | 5 | Weighted mode | 0.104 |  |  |  |  |  | -0.034 | 0.016 |
|  | MMP-1 | 32 | MR Egger | 0.022 | 0.006 | 0.176 | 34.574 | 0.13 | 0.259 | -0.079 | 0.033 |
|  |  | 32 | Weighted median | 0.241 |  |  |  |  |  | -0.032 | 0.027 |
|  |  | 32 | IVW | 0.036 |  |  | 36.789 | 0.16 | 0.219 | -0.043 | 0.021 |
|  |  | 32 | Simple mode | 0.873 |  |  |  |  |  | -0.008 | 0.048 |
|  |  | 32 | Weighted mode | 0.261 |  |  |  |  |  | -0.039 | 0.034 |
| UE | CXCL1 | 37 | MR Egger | 0.003 | -0.068 | 0.11 | 31.301 | 0 | 0.647 | 0.428 | 0.258 |
|  |  | 37 | Weighted median | 0.062 |  |  |  |  |  | 0.508 | 0.273 |
|  |  | 37 | IVW | 0.004 |  |  | 33.984 | 0 | 0.565 | 0.528 | 0.182 |
|  |  | 37 | Simple mode | 0.794 |  |  |  |  |  | 0.594 | 0.194 |
|  |  | 37 | Weighted mode | 0.056 |  |  |  |  |  | 0.056 | 0.056 |
|  | IL13 | 31 | MR Egger | 0.011 | 0.042 | 0.243 | 23.656 | 0 | 0.746 | -0.737 | 0.273 |
|  |  | 31 | Weighted median | 0.156 |  |  |  |  |  | -0.396 | 0.279 |
|  |  | 31 | IVW | 0.007 |  |  | 25.075 | 0 | 0.721 | -0.497 | 0.184 |
|  |  | 31 | Simple mode | 0.589 |  |  |  |  |  | -0.261 | 0.475 |
|  |  | 31 | Weighted mode | 0.488 |  |  |  |  |  | -0.301 | 0.429 |
|  | CDCP1 | 44 | MR Egger | 0.791 | 0.048 | 0.201 | 28.491 | 0 | 0.945 | 0.081 | 0.301 |
|  |  | 44 | Weighted median | 0.375 |  |  |  |  |  | 0.237 | 0.267 |
|  |  | 44 | IVW | 0.032 |  |  | 30.185 | 0 | 0.931 | 0.391 | 0.182 |
|  |  | 44 | Simple mode | 0.428 |  |  |  |  |  | 0.392 | 0.291 |
|  |  | 44 | Weighted mode | 0.544 |  |  |  |  |  | 0.183 | 0.311 |
|  | M-CSF | 38 | MR Egger | 0.234 | 0.011 | 0.814 | 34.126 | 0 | 0.558 | 0.356 | 0.293 |
|  |  | 38 | Weighted median | 0.197 |  |  |  |  |  | 0.367 | 0.284 |
|  |  | 38 | IVW | 0.033 |  |  | 34.182 | 0 | 0.602 | 0.408 | 0.192 |
|  |  | 38 | Simple mode | 0.558 |  |  |  |  |  | 0.301 | 0.211 |
|  |  | 38 | Weighted mode | 0.162 |  |  |  |  |  | 0.494 | 0.246 |
|  | NKR2B4 | 53 | MR Egger | 0.369 | 0.022 | 0.512 | 29.565 | 0 | 0.993 | 0.216 | 0.238 |
|  |  | 53 | Weighted median | 0.083 |  |  |  |  |  | 0.415 | 0.239 |
|  |  | 53 | IVW | 0.036 |  |  | 30.002 | 0 | 0.994 | 0.333 | 0.159 |
|  |  | 53 | Simple mode | 0.509 |  |  |  |  |  | 0.261 | 0.393 |
|  |  | 53 | Weighted mode | 0.278 |  |  |  |  |  | 0.328 | 0.299 |
