# Supplementary material for: Systemic Inflammatory Factors and Neuropsychiatric Disorders: A Bidirectional Mendelian Randomization Study
Source: Brain Behav. 2025 Apr 9;15(4):e70478. doi: 10.1002/brb3.70478 (PMC11979492; doi:10.1002/brb3.70478)

Exposure	Outcome	nSNP	MR_test	P_value	Pleiotropy_test	P.for.pleiotropy	Cochran.s.Q.test	I2	P.for.heterogeneity
AD	CDCP 1	44	MR Egger	0.042	0.002	0.751	45.021	0.07	0.347
	CDCP 1	44	Weighted median	0.018					
	CDCP 1	44	Inverse variance weighted	0.002			45.131	0.05	0.383
	CDCP 1	44	Simple mode	0.053					
	CDCP 1	44	Weighted mode	0.033					
	Axin−1	25	MR Egger	0.127	0.001	0.939	36.954	0.38	0.053
	Axin−1	25	Weighted median	0.023					
	Axin−1	25	Inverse variance weighted	0.008			36.964	0.35	0.054
	Axin−1	25	Simple mode	0.048					
	Axin−1	25	Weighted mode	0.068					
	IL8	38	MR Egger	0.024	−0.004	0.442	24.314	0	0.931
	IL8	38	Weighted median	0.032					
	IL8	38	Inverse variance weighted	0.013			24.92	0	0.935
	IL8	38	Simple mode	0.136					
	IL8	38	Weighted mode	0.031					
	MCP2	9	MR Egger	0.071	−0.007	0.56	5.222	0	0.633
	MCP2	9	Weighted median	0.022					
	MCP2	9	Inverse variance weighted	0.026			5.595	0	0.692
	MCP2	9	Simple mode	0.321					
	MCP2	9	Weighted mode	0.055					
	IL20	37	MR Egger	0.018	0.011	0.191	28.662	0	0.767
	IL20	37	Weighted median	0.103					
	IL20	37	Inverse variance weighted	0.033			30.443	0	0.731
	IL20	37	Simple mode	0.999					
	IL20	37	Weighted mode	0.088					
PD	SULT1A1	47	MR Egger	0.153	−0.001	0.929	52.412	0.14	0.209
	SULT1A1	47	Weighted median	0.146					
	SULT1A1	47	Inverse variance weighted	0.011			52.421	0.12	0.239
	SULT1A1	47	Simple mode	0.249					
	SULT1A1	47	Weighted mode	0.345					
	IL−15RA	31	MR Egger	0.399	−0.011	0.382	26.579	0	0.594
	IL−15RA	31	Weighted median	0.061					
	IL−15RA	31	Inverse variance weighted	0.014			27.367	0	0.604
	IL−15RA	31	Simple mode	0.555					
	IL−15RA	31	Weighted mode	0.042					
	CXCL1	37	MR Egger	0.071	−0.003	0.786	40.912	0.14	0.227
	CXCL1	37	Weighted median	0.055					
	CXCL1	37	Inverse variance weighted	0.015			40.999	0.12	0.261
	CXCL1	37	Simple mode	0.132					
	CXCL1	37	Weighted mode	0.029					
	MIP1a	40	MR Egger	0.014	−0.014	0.143	35.925	0	0.566
	MIP1a	40	Weighted median	0.024					
	MIP1a	40	Inverse variance weighted	0.031			38.165	0	0.508
	MIP1a	40	Simple mode	0.068					
	MIP1a	40	Weighted mode	0.006					
	S100−A12	45	MR Egger	0.056	0.009	0.364	42.104	0	0.511
	S100−A12	45	Weighted median	0.012					
	S100−A12	45	Inverse variance weighted	0.032			42.945	0	0.517
	S100−A12	45	Simple mode	0.063					
	S100−A12	45	Weighted mode	0.074					
	EIF4EBP1	36	MR Egger	0.232	0.003	0.745	31.191	0	0.606
	EIF4EBP1	36	Weighted median	0.282					
	EIF4EBP1	36	Inverse variance weighted	0.043			31.299	0	0.647
	EIF4EBP1	36	Simple mode	0.042					
	EIF4EBP1	36	Weighted mode	0.093					
	PD−L1	37	MR Egger	0.106	0.006	0.588	33.915	0	0.521
	PD−L1	37	Weighted median	0.062					
	PD−L1	37	Inverse variance weighted	0.047			34.213	0	0.554
	PD−L1	37	Simple mode	0.067					
	PD−L1	37	Weighted mode	0.118					
	CCL4	41	MR Egger	0.162	0.001	0.948	38.932	0	0.473
	CCL4	41	Weighted median	0.139					
	CCL4	41	Inverse variance weighted	0.048			38.936	0	0.518
	CCL4	41	Simple mode	0.031					
	CCL4	41	Weighted mode	0.171					
MS	MIP−1a	40	MR Egger	0.204	0.021	0.141	40.758	0.07	0.351
	MIP−1a	40	Weighted median	0.193					
	MIP−1a	40	Inverse variance weighted	0.001			43.202	0.1	0.296
	MIP−1a	40	Simple mode	0.014					
	MIP−1a	40	Weighted mode	0.031					
	TNF−beta	8	MR Egger	0.609	0.102	0.199	10.787	0.44	0.095
	TNF−beta	8	Weighted median	0.001					
	TNF−beta	8	Inverse variance weighted	0.004			14.529	0.52	0.053
	TNF−beta	8	Simple mode	0.048					
	TNF−beta	8	Weighted mode	0.826					
	CD40L	37	MR Egger	0.032	0.011	0.408	36.274	0.04	0.409
	CD40L	37	Weighted median	0.001					
	CD40L	37	Inverse variance weighted	0.023			37.001	0.03	0.423
	CD40L	37	Simple mode	0.203					
	CD40L	37	Weighted mode	0.002					
	IL1a	34	MR Egger	0.219	0.005	0.811	42.035	0.24	0.111
	IL1a	34	Weighted median	0.387					
	IL1a	34	Inverse variance weighted	0.033			42.111	0.22	0.133
	IL1a	34	Simple mode	0.859					
	IL1a	34	Weighted mode	0.613					
	Artemin	39	MR Egger	0.041	0.015	0.347	27.601	0	0.869
	Artemin	39	Weighted median	0.454					
	Artemin	39	Inverse variance weighted	0.034			28.508	0	0.868
	Artemin	39	Simple mode	0.366					
	Artemin	39	Weighted mode	0.588					
	CXC11	51	MR Egger	0.011	−0.026	0.092	67.854	0.28	0.058
	CXC11	51	Weighted median	0.064					
	CXC11	51	Inverse variance weighted	0.040			71.944	0.31	0.053
	CXC11	51	Simple mode	0.847					
	CXC11	51	Weighted mode	0.337					
ANX	CD40L	37	MR Egger	0.001	0.007	0.058	30.771	0	0.673
	CD40L	37	Weighted median	0.001					
	CD40L	37	Inverse variance weighted	0.001			35.436	0	0.495
	CD40L	37	Simple mode	0.462					
	CD40L	37	Weighted mode	0.001					
	M−CSF1	38	MR Egger	0.202	0.003	0.516	46.648	0	0.111
	M−CSF1	38	Weighted median	0.147					
	M−CSF1	38	Inverse variance weighted	0.006			47.206	0	0.121
	M−CSF1	38	Simple mode	0.043					
	M−CSF1	38	Weighted mode	0.249					
	CXL11	51	MR Egger	0.031	−0.002	0.539	42.221	0	0.743
	CXL11	51	Weighted median	0.056					
	CXL11	51	Inverse variance weighted	0.007			42.604	0	0.762
	CXL11	51	Simple mode	0.186					
	CXL11	51	Weighted mode	0.057					
	OPG	38	MR Egger	0.437	−0.005	0.179	19.754	0	0.987
	OPG	38	Weighted median	0.492					
	OPG	38	Inverse variance weighted	0.007			21.634	0	0.979
	OPG	38	Simple mode	0.401					
	OPG	38	Weighted mode	0.438					
	IL17C	38	MR Egger	0.793	−0.007	0.065	35.596	0	0.488
	IL17C	38	Weighted median	0.193					
	IL17C	38	Inverse variance weighted	0.011			39.904	0.07	0.342
	IL17C	38	Simple mode	0.241					
	IL17C	38	Weighted mode	0.298					
	TNFRSF9	43	MR Egger	0.109	−0.001	0.818	33.028	0	0.808
	TNFRSF9	43	Weighted median	0.055					
	TNFRSF9	43	Inverse variance weighted	0.011			33.082	0	0.836
	TNFRSF9	43	Simple mode	0.271					
	TNFRSF9	43	Weighted mode	0.171					
	CCL19	41	MR Egger	0.418	−0.004	0.348	36.338	0	0.592
	CCL19	41	Weighted median	0.091					
	CCL19	41	Inverse variance weighted	0.017			37.241	0	0.595
	CCL19	41	Simple mode	0.994					
	CCL19	41	Weighted mode	0.109					
	IL10RA	43	MR Egger	0.076	0.001	0.996	43.772	0.06	0.355
	IL10RA	43	Weighted median	0.645					
	IL10RA	43	Inverse variance weighted	0.017			43.772	0.04	0.396
	IL10RA	43	Simple mode	0.381					
	IL10RA	43	Weighted mode	0.292					
	IL12B	20	MR Egger	0.364	0.002	0.807	30.631	0.41	0.052
	IL12B	20	Weighted median	0.103					
	IL12B	20	Inverse variance weighted	0.021			30.736	0.38	0.053
	IL12B	20	Simple mode	0.465					
	IL12B	20	Weighted mode	0.105					
	IL24	29	MR Egger	0.503	−0.008	0.181	32.611	0.17	0.211
	IL24	29	Weighted median	0.552					
	IL24	29	Inverse variance weighted	0.025			34.896	0.2	0.173
	IL24	29	Simple mode	0.291					
	IL24	29	Weighted mode	0.752					
	S100−A12	45	MR Egger	0.189	0.001	0.901	58.794	0.27	0.055
	S100−A12	45	Weighted median	0.585					
	S100−A12	45	Inverse variance weighted	0.031			58.815	0.25	0.067
	S100−A12	45	Simple mode	0.409					
	S100−A12	45	Weighted mode	0.734					
	CD6	5	MR Egger	0.176	−0.002	0.738	2.654	0	0.448
	CD6	5	Weighted median	0.042					
	CD6	5	Inverse variance weighted	0.047			2.788	0	0.594
	CD6	5	Simple mode	0.677					
	CD6	5	Weighted mode	0.137					
DEP	VEGFA	42	MR Egger	0.091	0.001	0.745	45.192	0.11	0.264
	VEGFA	42	Weighted median	0.004					
	VEGFA	42	Inverse variance weighted	0.006			45.313	0.1	0.297
	VEGFA	42	Simple mode	0.513					
	VEGFA	42	Weighted mode	0.006					
	IL17C	38	MR Egger	0.224	−0.003	0.392	39.806	0.1	0.305
	IL17C	38	Weighted median	0.025					
	IL17C	38	Inverse variance weighted	0.006			40.635	0.09	0.313
	IL17C	38	Simple mode	0.975					
	IL17C	38	Weighted mode	0.045					
	CXCL10	45	MR Egger	0.065	0.001	0.605	38.712	0	0.658
	CXCL10	45	Weighted median	0.428					
	CXCL10	45	Inverse variance weighted	0.008			38.983	0	0.686
	CXCL10	45	Simple mode	0.272					
	CXCL10	45	Weighted mode	0.392					
	ADA	5	MR Egger	0.205	−0.001	0.834	2.085	0	0.555
	ADA	5	Weighted median	0.029					
	ADA	5	Inverse variance weighted	0.033			2.137	0	0.711
	ADA	5	Simple mode	0.809					
	ADA	5	Weighted mode	0.104					
	MMP−1	32	MR Egger	0.022	0.006	0.176	34.574	0.13	0.259
	MMP−1	32	Weighted median	0.241					
	MMP−1	32	Inverse variance weighted	0.036			36.789	0.16	0.219
	MMP−1	32	Simple mode	0.873					
	MMP−1	32	Weighted mode	0.261					
UE	CXCL1	37	MR Egger	0.003	−0.068	0.11	31.301	0	0.647
	CXCL1	37	Weighted median	0.062					
	CXCL1	37	Inverse variance weighted	0.004			33.984	0	0.565
	CXCL1	37	Simple mode	0.794					
	CXCL1	37	Weighted mode	0.056					
	IL13	31	MR Egger	0.011	0.042	0.243	23.656	0	0.746
	IL13	31	Weighted median	0.156					
	IL13	31	Inverse variance weighted	0.007			25.075	0	0.721
	IL13	31	Simple mode	0.589					
	IL13	31	Weighted mode	0.488					
	CDCP1	44	MR Egger	0.791	0.048	0.201	28.491	0	0.945
	CDCP1	44	Weighted median	0.375					
	CDCP1	44	Inverse variance weighted	0.032			30.185	0	0.931
	CDCP1	44	Simple mode	0.428					
	CDCP1	44	Weighted mode	0.544					
	M−CSF	38	MR Egger	0.234	0.011	0.814	34.126	0	0.558
	M−CSF	38	Weighted median	0.197					
	M−CSF	38	Inverse variance weighted	0.033			34.182	0	0.602
	M−CSF	38	Simple mode	0.558					
	M−CSF	38	Weighted mode	0.162					
	NKR2B4	53	MR Egger	0.369	0.022	0.512	29.565	0	0.993
	NKR2B4	53	Weighted median	0.083					
	NKR2B4	53	Inverse variance weighted	0.036			30.002	0	0.994
	NKR2B4	53	Simple mode	0.509					
	NKR2B4	53	Weighted mode	0.278					

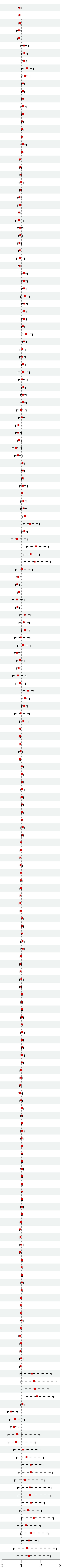

Supplement: Supplementary file 4 — Supplementary Materials. [file BRB3-15-e70478-s001.pdf]
